# The Effects of Concurrent Training Versus Aerobic or Resistance Training Alone on Body Composition in Middle-Aged and Older Adults: A Systematic Review and Meta-Analysis

**DOI:** 10.3390/healthcare13070776

**Published:** 2025-03-31

**Authors:** Mousa Khalafi, Shokoufeh Kheradmand, Aref Habibi Maleki, Michael E. Symonds, Sara K. Rosenkranz, Alexios Batrakoulis

**Affiliations:** 1 Department of Sport Sciences, Faculty of Humanities, University of Kashan, Kashan 87317-53153, Iran; 2 Department of Exercise Physiology, Faculty of Sport Sciences, University of Mazandaran, Babolsar 47416-13534, Iran; umz.kheradmand@gmail.com; 3 Physiology Research Center, Iran University of Medical Sciences, Tehran 14496-14535, Iran; habibimaleki.a@iums.ac.ir; 4 Centre for Perinatal Research, Academic Unit of Population and Lifespan Sciences, School of Medicine, University of Nottingham, Nottingham NG7 2UH, UK; michael.symonds@nottingham.ac.uk; 5 Department of Kinesiology and Nutrition Sciences, University of Nevada Las Vegas, Las Vegas, NV 89154, USA; sara.rosenkranz@unlv.edu; 6 Department of Physical Education and Sport Science, Democritus University of Thrace, 69100 Komotini, Greece; 7 Department of Physical Education and Sport Science, University of Thessaly, 42100 Trikala, Greece

**Keywords:** concurrent training, body composition, muscle mass, fat mass, older adults

## Abstract

**Introduction and Aim:** The beneficial effects of aerobic training (AT) on preventing excess fat mass, and of resistance training (RT) on skeletal muscle adaptation, are well established. However, the effects of concurrent training (CT) compared to AT or RT alone on body composition in middle-aged and older adults are less understood, and therefore, the focus of this meta-analysis. **Methods**: Three databases, including PubMed, Web of Science, and Scopus, were searched from inception to March 2024. Randomized trials were included if they compared CT versus either AT or RT, and included body composition measures such as fat mass, body fat percentage, waist circumference, visceral fat mass, lean body mass (LBM), muscle mass/volume, or muscle or muscle fiber cross-sectional area (CSA), in middle-aged (50 to <65 years) and older adults (≥65 years). Weighted mean differences (WMD) or standardized mean differences (SMD) and 95% confidence intervals (CIs) were calculated using random effects models. **Results**: A total of 53 studies involving 2873 participants were included. Overall, CT increased body weight and LBM significantly more, trending toward significantly larger increases in muscle mass and CSA, compared with AT alone. However, there were no significant differences between CT and RT alone, for body weight, BMI, body fat percentage, fat mass, waist circumference, or visceral fat mass. **Conclusions**: CT is as effective as AT for decreasing body fat measures and as effective as RT for increasing muscle mass in middle-aged and older adults, and it should be recommended accordingly.

## 1. Introduction

Aging is associated with progressive physiological changes impacting on body structure, function, health, and the risk of chronic disease and mortality [1]. One important consequence of aging is the progressive changes in body composition, with progressive loss of skeletal muscle and increased body fat, mainly in visceral depots [2,3,4,5,6]. In addition, after 30 years of age, muscle mass is lost at a rate of about 3–8% per annum, and accelerates after 50 years of age [6]. The age-related loss of skeletal muscle mass and function, called sarcopenia, is associated with mobility, loss of strength, fall-related injuries, hospitalization, and mortality [5,6,7,8,9]. Although there is a bidirectional relationship between sarcopenia and cardiovascular (CVDs) and metabolic diseases [10,11,12], sarcopenia is a specific clinical condition that can lead to insulin resistance and inflammation [10,11,12,13,14]. Along with skeletal muscle loss, age-related increases in adiposity and fat redistribution towards abdominal fat, and increased fat deposition in the liver, heart, and skeletal muscle [2,15], further contribute to age-related diseases [16,17].

Exercise training and physical activity are effective and safe non-pharmacological interventions for older adults that can improve cognitive function, cardiometabolic health, cardiorespiratory fitness, muscle mass, and muscular strength [18]. Despite the beneficial effects of resistance training (RT) on fat mass and visceral fat [19,20], aerobic-based exercise training (AT) is the most widely studied [21,22]. In contrast, RT is considered more important for increasing and maintaining muscle mass [23,24,25,26,27,28]. Given the simultaneous loss of skeletal muscle, rise in fat mass, and concomitant changes in body fat distribution with advancing age, a combination of AT and RT (concurrent training; CT) may optimize the benefits of exercise training [29,30,31,32,33]. Currently, previously published randomized trials have reported inconsistent results [34,35,36,37,38,39,40,41,42,43,44,45,46,47,48,49,50,51], and to our knowledge, there is no meta-analysis addressing the effects of CT versus either AT or RT alone on muscle and fat mass in older adults. One recent meta-analysis that examined muscle strength and cardiorespiratory fitness in middle-aged and elderly adults suggested that CT is as effective as either RT for improving muscular strength or AT for improving cardiorespiratory fitness [52]. Despite the potential additive effects of CT for disease prevention and athletic performance, there is some controversy around whether this mode of exercise has negative effects on muscle mass, strength and power, i.e., the interference effect [53,54,55]. Adding endurance training (aerobic training) to resistance training may impair the development of hypertrophy by inhibiting translational signaling downstream of Akt/mTOR and muscle protein synthesis [54,56,57]. In addition, a larger training volume may lead to chronic fatigue with CT compared to a single exercise [58].

One previous meta-analysis suggests that CT has negative effects on lower-body strength in trained, but not in untrained individuals [59], whilst another meta-analysis indicated that CT does not compromise muscle hypertrophy or maximal strength development, but the magnitude of gain may be attenuated [60]. Therefore, a comprehensive meta-analysis in middle-aged and older adults was necessary to investigate the potential effects of CT. Our hypothesis was that CT would be as effective as AT or RT alone for decreasing body fat mass and muscle mass, respectively. Secondly, CT would be more effective than AT for decreasing adiposity and increased muscle mass. We, therefore, investigated the effects of CT versus AT or RT alone on body composition in middle-aged (50 to <65 years) and older adults (≥65 years), and aimed to clarify the roles of age, BMI, and exercise moderators on the effectiveness of each intervention.

## 2. Methods

This systematic review and meta-analysis was conducted according to the Preferred Reporting Items for Systematic reviews and Meta-Analyses (PRISMA) guidelines and the Cochrane Handbook for Systematic Reviews of Interventions. The study protocol was established and registered in the International Prospective Register of Systematic Reviews (PROSPERO) with ID: CRD42024511774.

### 2.1. Search Strategy and Study Selection

A comprehensive literature search of three primary electronic databases including PubMed, Web of Science, and Scopus was conducted from inception to March 2024 to identify randomized trials that compared CT versus AT or RT alone. The search was conducted using four groups of keywords: aerobic training, resistance training, concurrent and older age. Results were limited to studies of human participants, publications written in the English language, and studies available in the searched databases. For additional studies, Google scholar and the reference lists of identified studies were manually searched to ensure that all relevant studies were included. The detailed search strategies for each database are presented in the Supplementary Table S1, and records identified from the database searches were imported into EndNote software (version 21) for further selection processes. After removing duplicate articles, study selection was conducted in two steps against inclusion and exclusion criteria. The first step included screening based on titles, abstracts, and key words. The second step included screening based on full texts. Comprehensive search and study section processes were performed independently by two authors (A.H.M. and S.K.) and any disagreements were resolved by discussion with another author (M.K.).

### 2.2. Identification and Selection Criteria

Studies were included if they were published in the English language in peer-reviewed journals and met the following PICOS criteria (population, intervention, comparator, outcomes, and study design).

Population: studies were included when they included middle-aged (50 to <65 years) and older adults (≥65 years), regardless of health status and biological sex. To increase the generalizability of the findings of the present study, older adults with and without chronic diseases were included in the meta-analysis; however, studies involving trained or athletic older adults were excluded. Intervention: Studies that involved CT, including a combination of AT and RT in the same or separate sessions, with intervention durations ≥ 2 weeks, were included. If balance and flexibility exercises were part of the CT protocol, they were also included. In addition, there were no limitations on the intensity, duration, or frequency of exercise sessions. However, CT protocols that used high-intensity interval training (HIIT) instead of AT were excluded. Comparator: Studies involving AT and/or RT alone were included; however, studies involving only a non-exercise control group, or other type of exercise such as HIIT, were excluded. Outcomes: Studies involving results for body composition including fat mass, body fat percentage (fat %), waist circumference, lean body mass (LBM) (fat free mass if LBM was not available), muscle mass or volume, and muscle and fiber cross-sectional area (CSA) as a main outcome, and body weight and body mass index (BMI) as secondary outcomes, were included. There were no limitations regarding measurement methods; therefore, studies that used dual-energy X-ray absorptiometry (DEXA), magnetic resonance imaging (MRI), computed tomography (CT) scans, bioelectrical impedance analysis (BIA), and skinfolds were included. Study design: Studies involving randomized parallel trials comparing CT versus AT and/or RT alone were included, whilst non-randomized trials were excluded.

### 2.3. Data Extraction and Synthesis

Data extraction for each study was performed independently by two authors (A.H.M. and S.K.) and checked by a third author (M.K.). The following data were extracted from each study: (1) study characteristics including the first author, year of publication, and study design; (2) participant characteristics including the number of participants and mean age, biological sex, BMI, and health status; (3) exercise training characteristics including type, mode, intensity, duration, and frequency; and (4) outcome variables and their measurement methods. For calculation of effect sizes, means and standard deviations (SD) at pre- and post-intervention were extracted where possible, and in the cases where these data were not available, mean changes (post values − pre values) and their SDs were extracted. If a study had more than one CT group, with an AT and/or RT group, each CT group was included separately, and the sample size of the opposite group was divided. When required, the data needed for calculating the effect sizes were extracted from figures using the Getdata Graph Digitizer software (version 2.26), and they were calculated from standard errors, medians, interquartile ranges, and confidence intervals [61,62,63]. For missing data, the corresponding authors were contacted if the article was published within the last 5 years. Despite these efforts, no responses were received from the three authors contacted.

### 2.4. Quality Assessment

The Physiotherapy Evidence Database Scale (PEDro) was used to assess the risk of bias and the overall quality of included studies. The PEDro Scale assesses bias using the following domains: 1. eligibility criteria were specified, 2. subjects were randomly allocated to groups, 3. allocation was concealed, 4. the groups were similar at baseline, 5. there was blinding of all subjects, 6. there was blinding of all therapists who administered the therapy, 7. there was blinding of all assessors who measured at least one key outcome, 8. measures of at least one key outcome were obtained from more than 85% of the subjects initially allocated to groups, 9. intention to treat (ITT) analysis, 10. the results of between-group statistical comparisons are reported, and 11. the study provides both point measures and measures of variability. However, two items including “5. there was blinding of all subjects and 6. there was blinding of all therapists who administered the therapy” were excluded from the scoring and evaluation due to the impossibility of performing them in exercise trials. Each study received a score ranging from zero to nine. The quality assessment for all included studies was completed by two independent authors (A.H.M. and S.K.)

### 2.5. Statistical Analysis

For each outcome, meta-analysis was performed to compare CT with either AT or RT only using pre- and post-intervention mean and SD values, or mean changes and their SDs and sample sizes. Standardized mean differences (SMD) or weighted mean differences (WMD) and 95% confidence intervals were calculated using random effects models to determine the effect sizes. WMD was used when the data were reported based on the same units, and SMD was used when the data were reported as more than one measurement unit. When there were more than 10 interventions for each outcome, subgroup analyses were performed based on age (middle-aged: 50 to <65 years and older adults: ≥65 years), biological sex (male, female, or both), BMI (obese: BMI ≥ 30 kg·m^2^ and non-obese BMI < 30 kg·m^2^), intervention duration (medium-term: <24 weeks and long-term: ≥24 weeks), and type of CT (within the same session or as separate sessions). Interpretation of SMDs was performed based on Cochrane guidelines as follows: <0.2 (trivial), 0.2 to <0.4 (small), 0.5 to <0.8 (moderate), and ≥0.8 (large). Heterogeneity amongst included studies was assessed using the Q statistic, which was considered significant at *p* < 0.05, and I^2^ statistics, which were interpreted as follows: 25% (low), 50% (moderate), and 70% (high) heterogeneity. Publication bias was assessed using visual interpretation of funnel plots and the Egger’s test was used as a secondary determinant. The trim and fill method for correction of publication bias was used when visual interpretation of funnel plots indicated bias was present [64]. In addition, sensitivity analyses were performed by removing studies with less-reliable measurement methods including BIA and skinfolds and studies with unmatched exercise volumes (between CT and AT/RT). All analyses were performed using comprehensive meta-analysis version 3 (CMA3) software.

## 3. Results

### 3.1. Search Results

**Figure 1:**
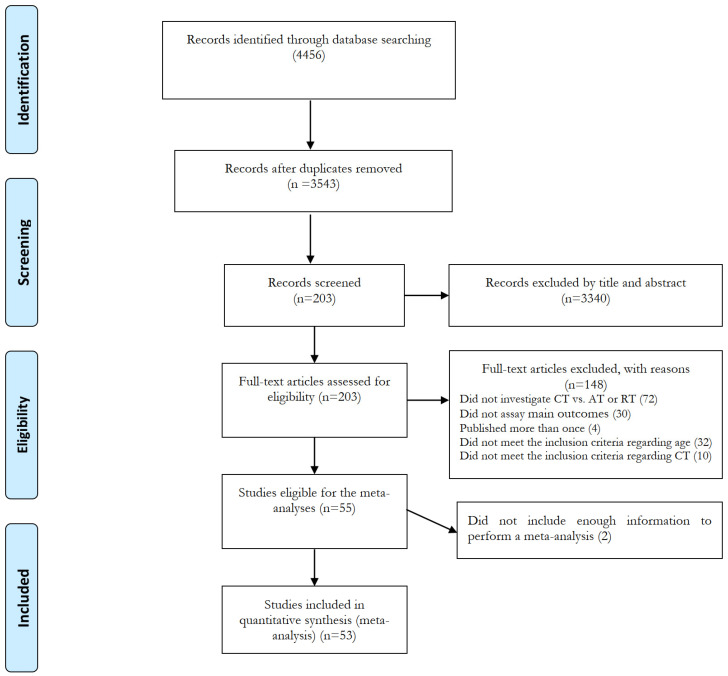
Flow diagram of systematic literature search.

The initial search yielded 4456 records, of which 3543 remained after removing duplicates. After the first step of screening based on titles, abstracts, and key words, 3340 articles were excluded, and subsequently, 203 articles were screened (second step) based on the full-texts against inclusion and exclusion criteria. Finally, 150 articles were excluded for reasons presented in Figure 1, and 53 articles met all inclusion criteria and were, therefore, included in the meta-analysis [34,35,36,37,38,39,40,41,42,43,44,45,46,47,48,49,50,65,66,67,68,69,70,71,72,73,74,75,76,77,78,79,80,81,82,83,84,85,86,87,88,89,90,91,92,93,94,95,96,97,98,99,100].

### 3.2. Study Characteristics

In total, 2873 middle-aged (50 to <65 years) and older adults (≥65 years) were included in the meta-analysis. The mean ages and BMIs of participants ranged from 51 [72] to 82 [100] years old, and 23 [48] to 35 [91] kg·m^2^. The sample sizes of studies varied, with a range from 15 [92] to 208 [39]. For the current meta-analysis, middle-aged (50 to <65 years) and older adults (≥65 years) were included, regardless of their health status. Participants with a wide range of health statuses and chronic diseases such obesity, type 2 diabetes, and cardiovascular diseases were included. The details of the participant characteristics are provided in Table 1. Intervention durations ranged from 6 weeks [80] to 12 months [65], whereas frequencies of sessions per week ranged from 2 to 5, with 3 sessions used in the most of studies. Most of the studies used supervised exercise sessions, and CT that included combined AT and RT in the same sessions was used in a majority of studies. The details of the training protocols used in each study are summarized in Table 2, while the overall quality of included studies is presented in the Supplementary Table S2. PEDro scores of the included studies ranged from 4 to 9.

### 3.3. Meta-Analysis

#### 3.3.1. CT vs. AT

**Figure 2:**
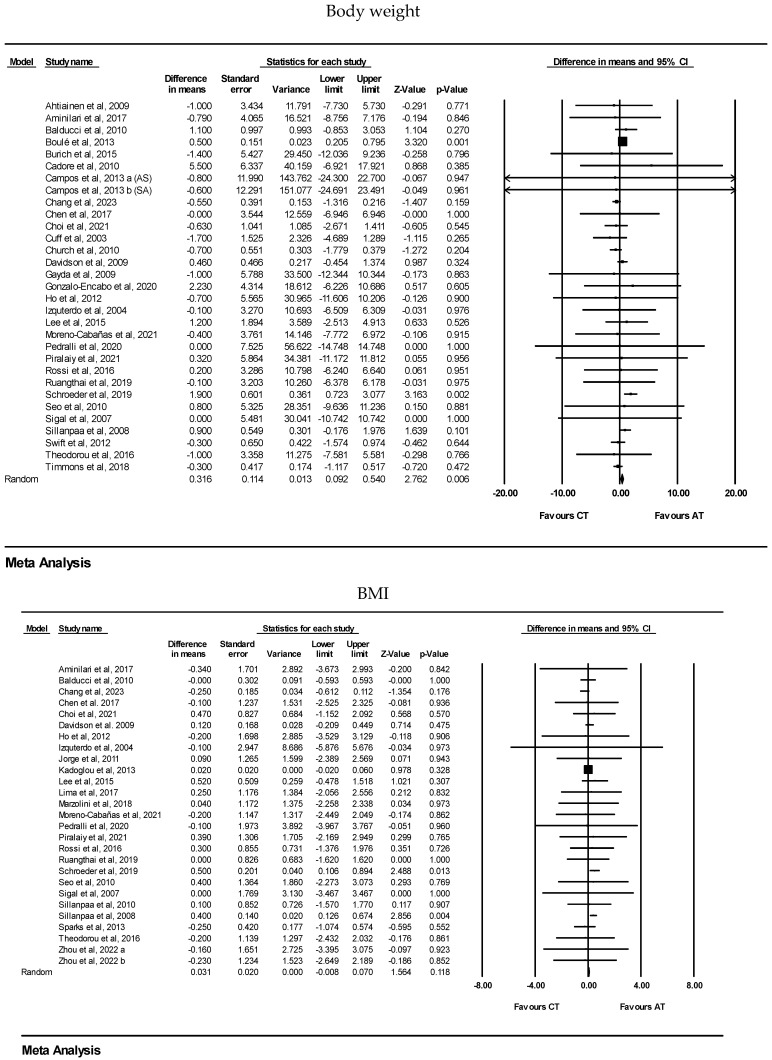

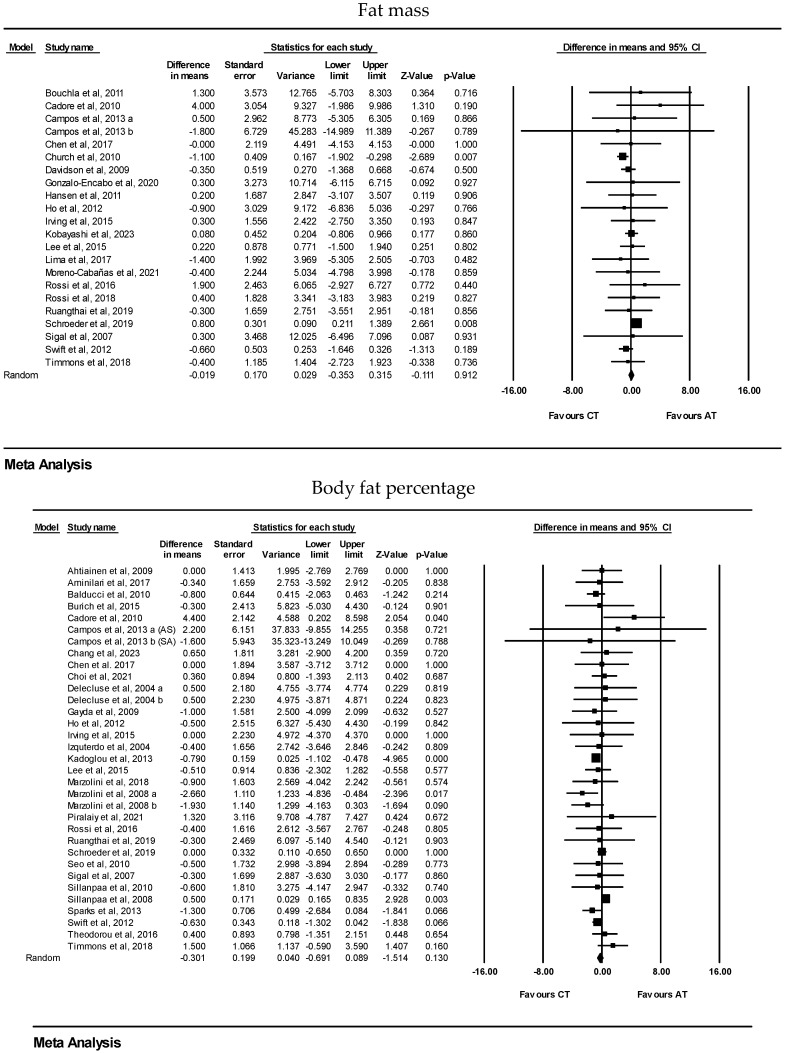

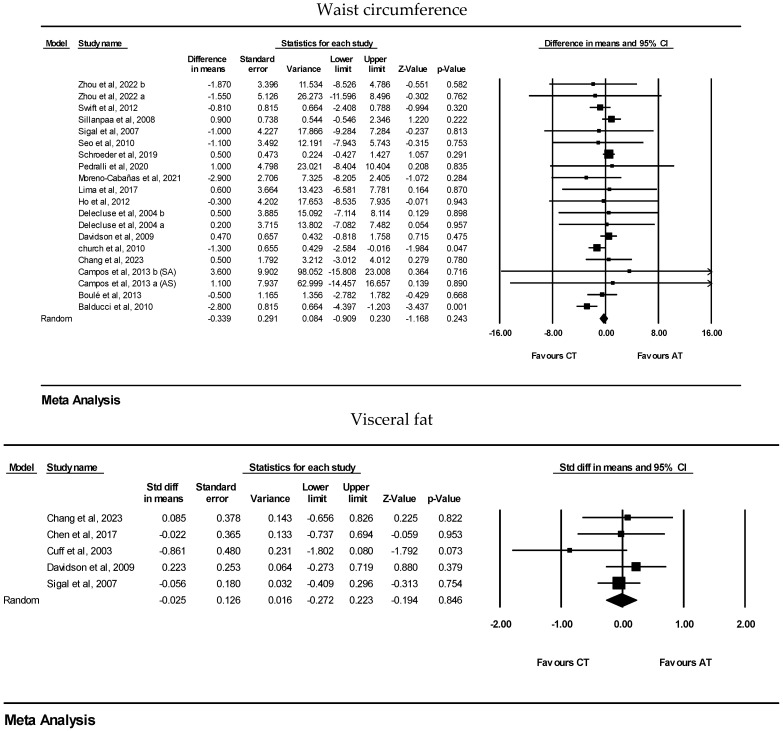

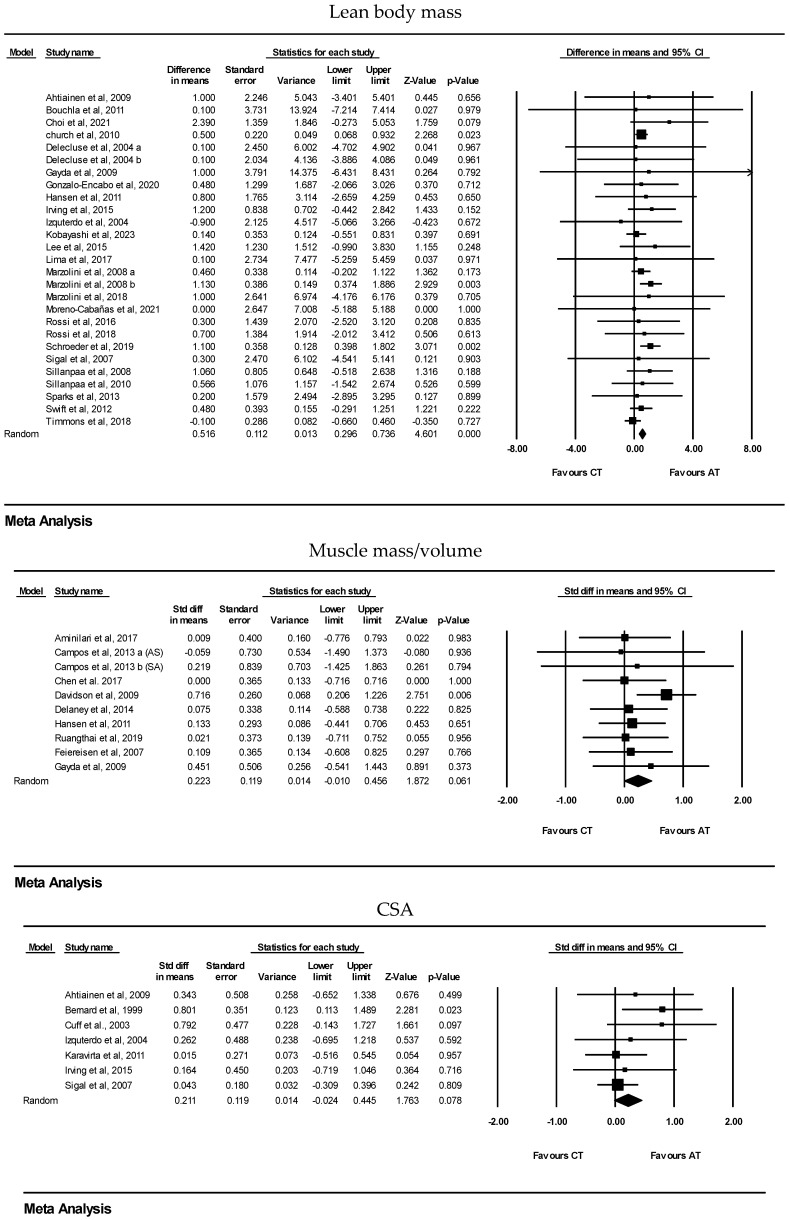
Forest plots of the effects of concurrent training (CT) versus aerobic training (AT) on body weight, BMI, fat mass, fat percentage, waist circumference, visceral fat, lean body mass, muscle mass/volume, and CSA. Data are reported as WMD (95% confidence limits) or SMD (95% confidence limits). WMD: weighted mean difference. SMD: standardized mean difference.

CT increased body weight [WMD: 0.31 kg (95% CI: 0.09 to 0.54), *p* = 0.006; 31 trials] and lean body mass [WMD: 0.51 kg (95% CI: 0.29 to 0.73), *p* = 0.001; 27 trials] significantly more than AT alone. However, there were no significant differences between CT and AT alone for changes in BMI [WMD: 0.03 kg·m^2^ (95% CI: −0.00 to 0.07), *p* = 0.11; 27 trials], body fat% [WMD: −0.30% (95% CI: −0.69 to 0.08), *p* = 0.13; 33 trials], fat mass [WMD: −0.01 kg (95% CI: −0.35 to 0.31), *p* = 0.91; 22 trials], waist circumference [WMD: −0.33 cm (95% CI: −0.90 to 0.23), *p* = 0.24; 20 trials], visceral fat mass [SMD: −0.02 (95% CI: −0.27 to 0.22), *p* = 0.84; 5 trials], muscle mass/volume [SMD: 0.22 (95% CI: −0.01 to 0.45), *p* = 0.06; 10 trials], and CSA [SMD: 0.21 (95% CI: −0.02 to 0.44), *p* = 0.07; 7 trials], (Figure 2 and Table 3). In addition, following sensitivity analysis by removing studies with unmatched exercise volumes between the two training interventions, there were no significant differences between CT and AT alone for body weight [WMD: 0.18 kg (95% CI: −0.38 to 0.76), *p* = 0.52; 14 trials], BMI [WMD: 0.02 kg·m^2^ (95% CI: −0.01 to 0.06), *p* = 0.20; 14 trials], fat mass [WMD: −0.16 kg (95% CI: −0.96 to 0.63), *p* = 0.67; 9 trials], waist circumference [WMD: −0.71 cm (95% CI: −1.75 to 0.32), *p* = 0.17; 11 trials], visceral fat mass [SMD: −0.11 (95% CI: −0.67 to 0.45), *p* = 0.70; 3 trials], muscle mass/volume [SMD: 0.25 (95% CI: −0.05 to 0.56), *p* = 0.10; 5 trials], and CSA [SMD: 0.21 (95% CI: −0.02 to 0.44), *p* = 0.07; 7 trials]. However, CT increased lean body mass [WMD: 0.45 kg (95% CI: 0.14 to 0.77), *p* = 0.004; 10 trials] and decreased body fat% [WMD: −0.54% (95% CI: −0.85 to −0.23), *p* = 0.001; 12 trials] significantly more than AT alone. Also, when sensitivity analyses were conducted by removing studies with less reliable measurement methods, CT decreased fat mass [WMD: −0.47 kg (95% CI: −0.90 to 0.04), *p* = 0.03; 11 trials] and increased lean body mass [WMD: 0.43 kg (95% CI: 0.19 to 0.67), *p* = 0.001; 15 trials] significantly more than AT alone. However, there were no significant differences between CT and AT alone for body fat% [WMD: −0.53% (95% CI: −1.31 to −0.24), *p* = 0.17; 11 trials] or muscle mass/volume [SMD: 0.30 (95% CI: −0.02 to 0.63), *p* = 0.06; 4 trials].

#### 3.3.2. CT vs. RT

**Figure 3:**
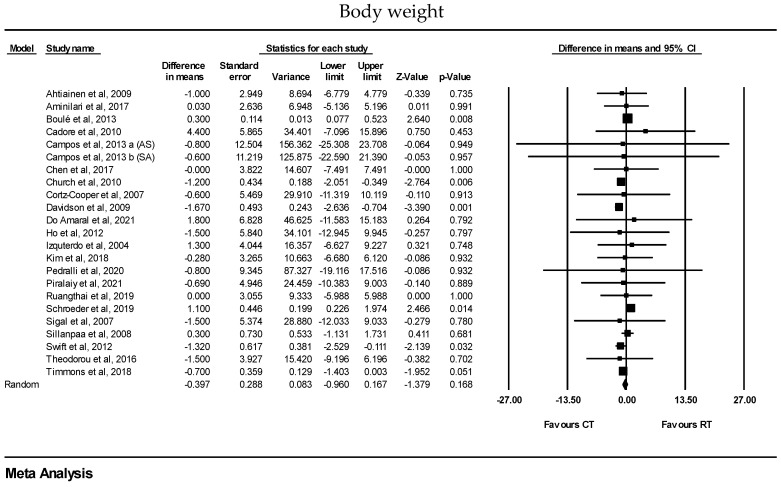

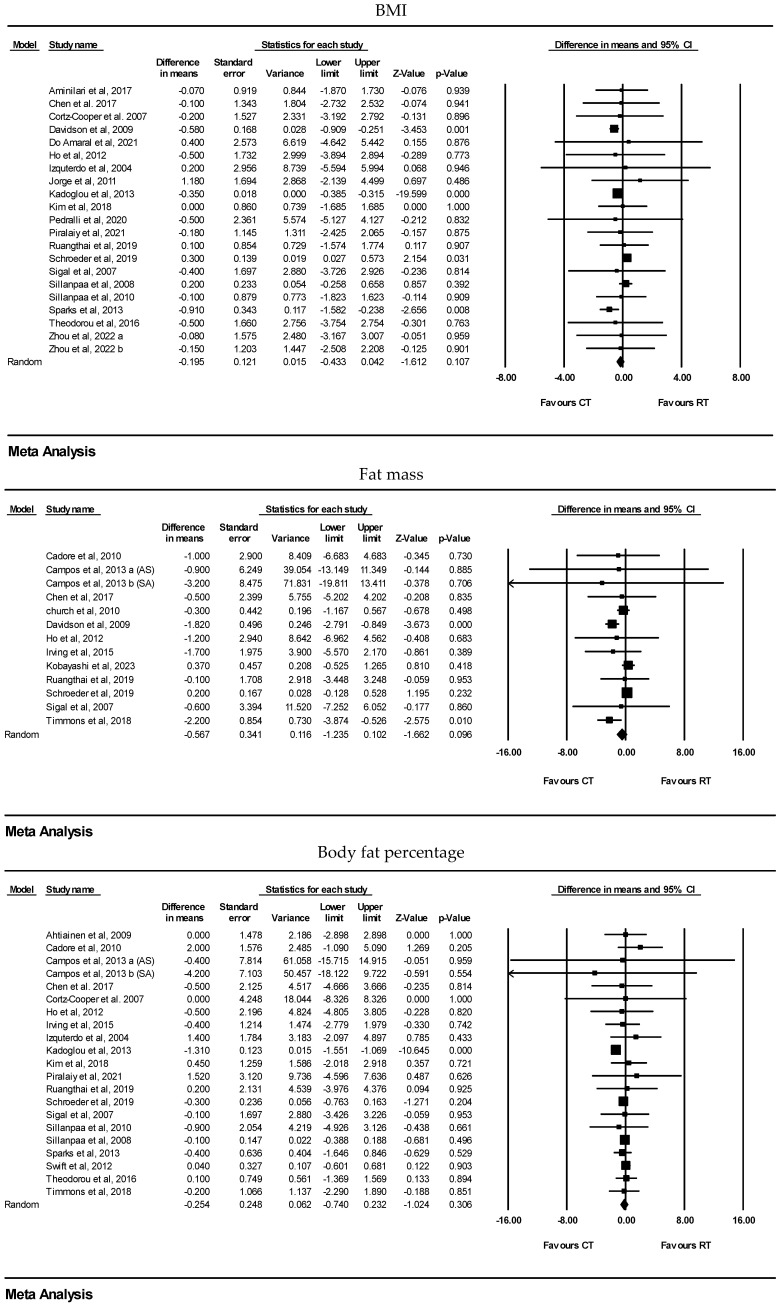

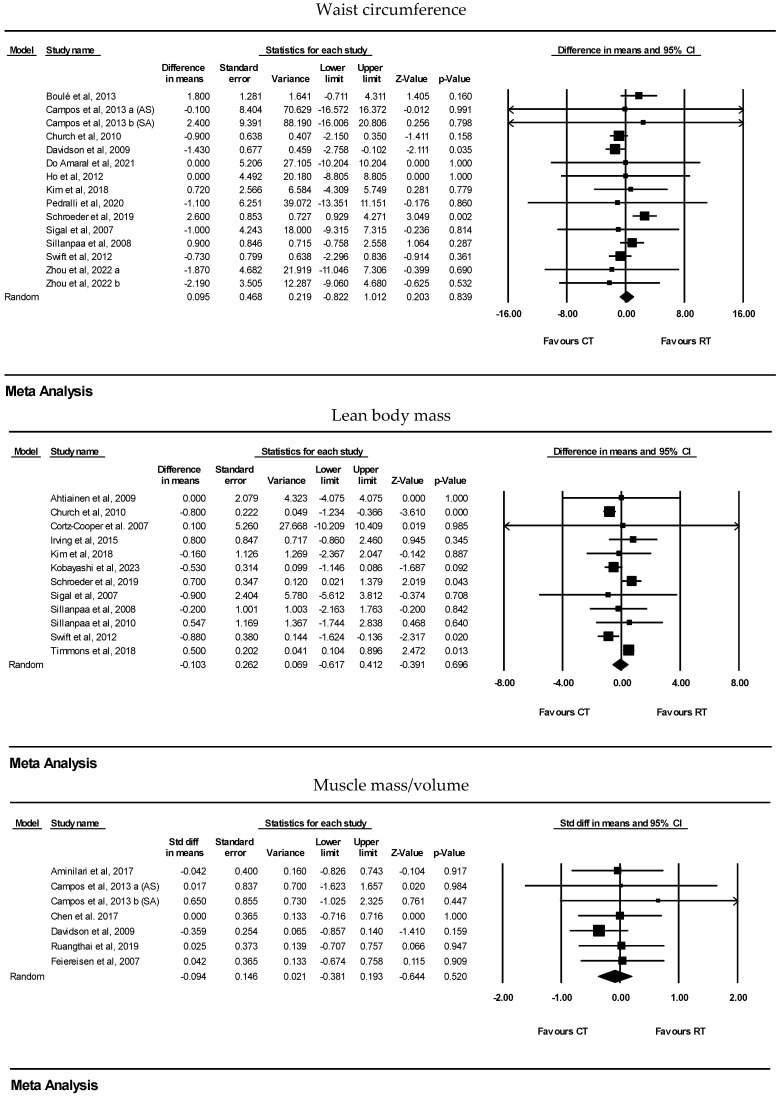

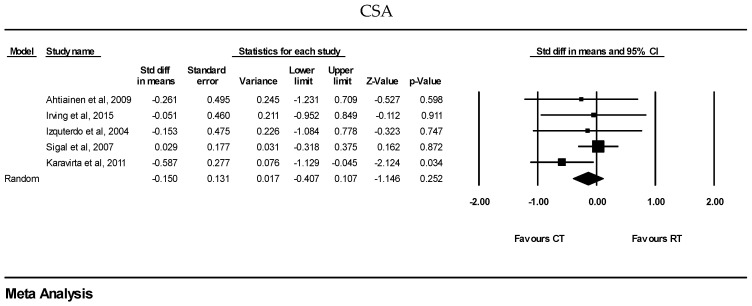
Forest plots of the effects of concurrent training (CT) versus resistance training (RT) on body weight, BMI, fat mass, fat percentage, waist circumference, lean body mass, muscle mass/volume, and CSA. Data are reported as WMD (95% confidence limits) or SMD (95% confidence limits). WMD: weighted mean difference; SMD: standardized mean difference.

There were no significant differences between CT and RT alone for changes in body weight [WMD: −0.39 kg (95% CI: −0.96 to 0.16), *p* = 0.16; 23 trials], BMI [WMD: −0.19 kg·m^2^ (95% CI: −0.43 to 0.04), *p* = 0.10; 21 trials], body fat% [WMD: −0.25% (95% CI: −0.74 to 0.23), *p* = 0.36; 21 trials], fat mass [WMD: −0.56 kg (95% CI: −1.23 to 0.10), *p* = 0.09; 13 trials], waist circumference [WMD: 0.09 cm (95% CI: −0.82 to 1.01), *p* = 0.83; 15 trials], lean body mass [WMD: −0.10 kg (95% CI: −0.61 to 0.41), *p* = 0.69; 12 trials], muscle mass/volume [SMD: −0.09 (95% CI: −0.38 to 0.19), *p* = 0.52; 7 trials], or CSA [SMD: −0.15 (95% CI: −0.40 to 0.10), *p* = 0.25; 5 trials] (Figure 3 and Table 3). In addition, sensitivity analyses conducted by removing unmatched exercise volumes between the two intervention arms showed that there were no significant differences between CT and RT alone for body weight [WMD: −0.26 kg (95% CI: −1.27 to 0.75), *p* = 0.61; 8 trials], BMI [WMD: −0.17 kg·m^2^ (95% CI: −0.57 to 0.22), *p* = 0.38; 11 trials], body fat% [WMD: −0.59% (95% CI: −1.27 to 0.08), *p* = 0.08; 7 trials], fat mass [WMD: −0.37 kg (95% CI: −1.19 to 0.44), *p* = 0.36; 5 trials], waist circumference [WMD: 0.35 cm (95% CI: −1.67 to 2.37), *p* = 0.73; 7 trials], lean body mass [WMD: 0.08 kg (95% CI: −0.78 to 0.95), *p* = 0.84; 4 trials], and muscle mass/volume [SMD: 0.02 (95% CI: −0.39 to 0.43), *p* = 0.91; 3 trials]. Moreover, when sensitivity analyses were conducted by removing studies with less reliable measurement methods, there were no significant differences between CT and RT alone for body fat% [WMD: −0.10% (95% CI: −0.35 to 0.15), *p* = 0.43; 8 trials], fat mass [WMD: −0.92 kg (95% CI: −1.93 to 0.08), *p* = 0.07; 6 trials], lean body mass [WMD: −0.23 kg (95% CI: −0.83 to 0.36), *p* = 0.44; 12 trials], and muscle mass/volume [SMD: −0.22 (95% CI: −0.63 to 0.18), *p* = 0.27; 2 trials].

### 3.4. Heterogeneity

CT vs. AT: There was significant heterogeneity amongst studies for changes in body fat% (I^2^ = 38.91, *p* = 0.01). However, there was no significant heterogeneity amongst included studies for changes in body weight (I^2^ = 0.00, *p* = 0.67), BMI (I^2^ = 0.00, *p* = 0.89), fat mass (I^2^ = 0.00, *p* = 0.52), lean body mass (I^2^ = 0.00, *p* = 0.95), waist circumference (I^2^ = 9.05, *p* = 0.34), visceral fat mass (I^2^ = 2.49, *p* = 0.39), or muscle mass/volume (I^2^ = 0.00, *p* = 0.80), or CSA (I^2^ = 0.00, *p* = 0.44).

CT vs. RT: There was significant heterogeneity amongst included studies for body weight (I^2^ = 46.94, *p* = 0.007), BMI (I2 = 39.81, *p* = 0.03), fat mass (I^2^ = 49.22, *p* = 0.02), body fat% (I^2^ = 64.90, *p* = 0.001), lean body mass (I^2^ = 65.34, *p* = 0.001). Meanwhile, there was not significant heterogeneity amongst included studies for waist circumference (I^2^ = 31.40, *p* = 0.11) or muscle mass/volume (I2 = 0.00, *p* = 0.90), or CSA (I2 = 0.00, *p* = 0.46).

### 3.5. Publication Bias

CT vs. AT: Visual interpretation of funnel plots suggested publication bias for body weight, BMI, body fat%, fat mass, and lean body mass, but the Egger’s tests did not confirm bias for body weight (*p* = 0.42), BMI (*p* = 0.32), body fat% (*p* = 0.99), fat mass (*p* = 0.96), lean body mass (*p* = 0.43), waist circumference (*p* = 0.65), or muscle mass/volume (*p* = 0.30), or CSA (*p* = 0.14). Both funnel plots and Egger’s tests did not suggest publication bias for visceral fat (*p* = 0.48). The trim and fill method indicated missing studies from the right and left side of the funnel plot, and after including those missing studies, the effect sizes were presented for the following factors: body weight [WMD: 0.33 kg (95% CI: 0.10 to 0.55), six trials from the right side of the mean], BMI [WMD: 0.03 kg·m^2^ (95% CI: −0.00 to 0.06), one trial from the left side of the mean], body fat% [WMD: −0.40% (95% CI: −0.81 to −0.00), three trials from the left side of the mean], fat mass [WMD: −0.03 kg (95% CI: −0.36 to 0.30), one trial from the left side of the mean], lean body mass [WMD: 0.49 kg (95% CI: 0.27 to 0.71), two trials from the left side of the mean], waist circumference [WMD: −0.31 cm (95% CI: −0.85 to 0.21), one trial from the left side of the mean], muscle mass/volume [SMD: 0.28 (95% CI: 0.07 to 0.49), three trials from the right side of the mean], and CSA [SMD: 0.07 (95% CI: −0.21 to 0.36), three trials from the left side of the mean].

CT vs. RT: Visual interpretation of funnel plots suggested publication bias for BMI, body fat%, fat mass, and lean body mass, but the Egger’s tests did not confirm bias for BMI (*p* = 0.25), body fat% (*p* = 0.14), fat mass (*p* = 0.11), lean body mass/volume (*p* = 0.94), waist circumference (*p* = 0.78), or CSA (*p* = 0.58). Both the funnel plot and Egger’s test suggested publication bias for muscle mass (*p* = 0.04). Furthermore, both the funnel plot and Egger’s test did not suggest publication bias for body weight (*p* = 0.24). The trim and fill method indicated missing studies from the right and left side of the plot, and after adding the missing studies, the effect sizes were presented for the following factors: BMI [WMD: −0.40 kg·m^2^ (95% CI: −0.64 to −0.16), seven trials from the left side of the funnel plot], body fat% [WMD: −0.38% (95% CI: −0.83 to 0.07), five trials from the left side of the funnel plot], fat mass [WMD: 0.10 kg·m^2^ (95% CI: −0.63 to 0.83), seven trials from the right side of the funnel plot], lean body mass [WMD: −0.16 kg (95% CI: −0.66 to 0.33), one trial from the left side of the funnel plot], waist circumference [WMD: 0.13 cm (95% CI: −0.72 to 0.99), two trials from the right side of the funnel plot], muscle mass/volume [SMD: −0.14 (95% CI: −0.41 to 0.11), two trials from the left side of mean], and CSA [SMD: 0.01 (95% CI: −0.28 to 0.31), three trials from the left side of the mean].

### 3.6. Subgroup Analysis

Subgroup analyses revealed that several factors, including session type (same session or separate), biological sex (male, female, or both), age (middle-aged or older adults), intervention duration (medium-term: <24 weeks and long-term: ≥24 weeks), and BMI (obesity: BMI ≥ 30 kg·m^2^ and, normal weight or overweight BMI < 30 kg·m^2^), served as key moderators influencing the effects of CT compared to AT and RT.

CT vs. AT: As compared with AT alone, CT induced significantly larger increases in body weight in middle-aged adults (WMD = 0.37 kg, *p* = 0.003), with medium-term intervention durations (WMD = 0.37 kg, *p* = 0.003), and when CT was performed in the same session (WMD = 0.35, *p* = 0.004). In addition, CT induced significantly larger increases in BMI for males (WMD = 0.39 kg·m^2^, *p* = 0.005), adults without obesity (WMD = 0.25 kg·m^2^, *p* = 0.008), and medium-term intervention durations (WMD = 0.24 kg·m^2^, *p* = 0.009), and when CT was performed in separate sessions (WMD = 0.32 kg·m^2^, *p* = 0.009) when compared with AT alone. CT induced significantly larger decreases in body fat% for middle-aged adults (WMD = −0.43%, *p* = 0.04), when both males and females were included (WMD = −0.66%, *p* = 0.001), in adults with obesity (WMD = −0.61%, *p* = 0.001), and with long-term intervention durations (WMD = −0.79%, *p* = 0.001) when compared with AT alone. Meanwhile, there were significantly larger increases in body fat% for males (WMD = 0.49%, *p* = 0.003) and medium-term intervention durations (WMD = 0.36%, *p* = 0.01). CT induced significantly larger increases in fat mass following medium-term intervention durations (WMD = 0.61 kg, *p* = 0.01) and significantly larger decreases following long-term intervention durations (WMD = −0.53 kg, *p* = 0.02) when compared with AT alone. For lean body mass, CT induced significantly larger increases in middle-aged adults (WMD = 0.59 kg, *p* = 0.001) when both males and females were included (WMD = 0.48 kg, *p* = 0.001), in adults with obesity (WMD = 0.61 kg, *p* = 0.001) and without obesity (WMD = 0.42 kg, *p* = 0.005), following both medium-term (WMD = 0.51 kg, *p* = 0.006) and long-term intervention durations (WMD = 0.51 kg, *p* = 0.001), and when CT was performed both as separate sessions (WMD = 0.69 kg, *p* = 0.001) or within the same sessions (WMD = 0.38 kg, *p* = 0.02) when compared with AT alone.

CT vs. RT: CT induced significantly larger decreases in body weight for older adults (WMD = −0.98 kg, *p* = 0.001) and long-term intervention durations (WMD = −1.37 kg, *p* = 0.001), and significantly larger decreases in BMI for older adults (WMD = −0.51 kg·m^2^, *p* = 0.001) and with long-term intervention duration (WMD = −0.35 kg·m^2^, *p* = 0.001), when compared with RT alone. However, CT induced significantly larger increases in BMI with medium-term intervention duration (WMD = 0.24 kg, *p* = 0.03) when compared with RT alone. CT induced significantly larger decreases in fat mass for older adults (WMD = −1.75 kg, *p* = 0.001), significantly larger increases in lean body mass for older adults (WMD = 0.49 kg, *p* = 0.01) and with medium-term intervention durations (WMD = 0.52 kg, *p* = 0.002), and significant decreases with long-term intervention durations (WMD = −0.74 kg, *p* = 0.001) when compared with RT alone. CT induced significantly larger decreases in waist circumference in older adults (WMD = −1.27 cm, *p* = 0.04) and with long-term intervention durations (WMD = −1.04 cm, *p* = 0.009) and significantly larger increases with medium-term intervention durations (WMD = 1.52 cm, *p* = 0.003) when compared with RT alone.

## 4. Discussion

The current meta-analysis aimed to clarify the effects of CT on body composition when compared to AT or RT alone in middle-aged (50 to <65 years) and older adults (≥65 years). These findings support our hypothesis that CT is significantly more effective than AT for increasing body weight [WMD: 0.31 kg] and lean body mass [WMD: 0.51 kg]. However, there were no significant differences between CT and AT regarding changes in BMI, body fat%, and waist circumference. Additionally, our results indicate that CT is no less effective than RT for increasing lean body mass and muscle mass, again aligning with our hypothesis regarding its non-inferiority. We observed no significant differences between CT and RT for changes in body weight or lean body mass, emphasizing the benefits of CT without compromising the advantages of RT. In light of the demographic shifts and increasing numbers of middle-aged and older adults, the significance of addressing strategies for managing sarcopenia is critical. CT can be positioned as a key strategy for maintaining and increasing body weight, specifically, muscle mass, and for improving body composition in this age group. However, we acknowledge that the effects of CT may vary across ages and intervention durations, suggesting the need for a nuanced interpretation of these outcomes.

Although the popularity and health benefits of exercise training, particularly for age-related increases in adiposity are well documented, in the current literature, there is limited meta-analytical evidence regarding the effects of CT versus AT and/or RT alone in older adults for body composition outcomes [101]. The prevalence of obesity among older adults and age-related changes in total and regional fat distribution are associated with increased risk of CVD [2,102,103]. We show that CT provides similar effects to AT and/or RT on adiposity markers including fat mass, body fat%, waist circumference, and visceral fat. Several meta-analyses have clarified the role that exercise mode plays on adiposity, such as reducing subcutaneous or visceral fat, with AT having the greatest effect [22,104]. A network meta-analysis ranked AT as best for improving body weight, BMI, and WC [105], although in some, the greater effects with AT may be due to the comparison used. In the present meta-analysis, “head-to-head” trials comparing CT with AT alone and/or RT alone indicate significant differences not found previously when CT was compared with AT alone [105], whilst we did not show any greater adiposity loss following CT as compared with RT alone. Despite the central role of RT on increasing muscle mass, it also reduced fat mass, as shown in two separate meta-analyses [19,105]. While the current meta-analysis was not designed to determine the mechanisms by which exercise effects on adiposity, these include increasing energy expenditure and regulating appetite [20,106,107,108]. However, the timing and volume of exercise may be important moderators for CT adaptations [109]. Among the included studies, these moderators were often not clearly investigated or reported, which limited our analyses. Nevertheless, the results of the sensitivity analyses when studies included the same exercise volumes between CT and AT arms suggest that the results are reliable.

Age-related muscle sarcopenia is common in older adults and leads to increased risks of CVD and metabolic disorders [8,110,111]. RT is a widely accepted exercise for both enhancing and maintaining muscle mass in people with different health conditions across all ages. Even if effect sizes are small, several meta-analyses have confirmed the potential positive role of RT for enhancing and maintaining muscle mass in old and very old adults [24,112]. However, the effect of CT versus AT alone and/or RT alone has rarely been studied in older adults, but we find that CT is as effective as RT, and superior to AT for increasing muscle mass, as reported by Schumann et al. [60], who indicate that CT does not compromise muscle hypertrophy. Wilson and colleagues [113] found significant differences between CT and AT, but not between CT and RT; although adding AT to RT is thought to create an “interference effect” on muscular strength and hypertrophy [53,54], that does not appear to be the case in the middle-aged and older adults. Incorporating CT into sarcopenia management may include practical applications in rehabilitation centers, senior fitness programs, and community-based initiatives for middle-aged and older adults. Applications could include activities that combine resistance training with aerobic exercises, which can enhance quality of life and activities of daily living, reduce cardiovascular disease risk, and effectively manage weight. We previously reported that improved muscular strength was not compromised by CT in middle-aged and older adults [52], with the magnitude of the “interference effect” with CT dependent on the subjects’ characteristics, especially their training status, age, biological sex, mode of AT, muscle fiber types, and outcomes assessed [59,60,114]. In particular, negative effects following CT have been reported in men, who regularly exercised [59,60,114]. It has been suggested that larger volumes and the timing of CT may affect the muscle mass outcomes versus RT alone due to the potential for fatigue [58]. Unequal volumes of CT and RT is an important limitation within included studies and, therefore, these results should be considered with caution. Furthermore, it is essential to briefly acknowledge the role of dietary factors in conjunction with exercise interventions, particularly regarding protein intake. Dietary protein is particularly important in middle-aged and older adults for maintaining an anabolic state, and it may help reduce the rate of sarcopenia in individuals of advanced age [115]. Although there is limited evidence linking high protein intake to an increased risk of impaired kidney function in healthy individuals, older adults should exercise caution, as renal function tends to decline with age [116,117]. On the other hand, while activation of the mTORC1 pathway is essential for muscle protein synthesis, caloric/protein restriction may exert protective effects against sarcopenia through partial mTORC1 inhibition and autophagy induction [118]. These seemingly contradictory findings may, at least in part, be explained by the presence of mTORC1-independent signaling pathways that influence muscle protein synthesis [118] and degradation, as well as the differential roles of the mTORC1/AMPK pathways throughout the lifespan [119]. Several moderators including age, BMI, biological sex, intervention duration, and type of CT may contribute to its effects on body composition, which tend to be greater in middle-aged compared with older adults (≥65 years). The longer an intervention, the greater the benefit for reduced weight and waist circumference, which may be modified by age. Aging is a risk factor for cardiometabolic diseases that are associated with increased adiposity, especially visceral, for which long-term weight loss is difficult [52]. CT could promote long-term benefits in the elderly, as it integrates both aerobic and resistance training elements, promoting overall health and functional capacity. In our meta-analysis, the limited number of studies on muscle mass and CSA outcomes did not allow us to perform subgroup analyses, and, therefore, only lean body mass was investigated. Age and intervention duration moderated the effect of CT that increased lean body mass in medium-term-duration studies, compared to longer-term interventions, when it actually decreased. Comparisons with previous meta-analyses is challenging, as these were mainly focused on athletes and trained adults [60,113,120]. However, when considering muscle mass hypertrophy, several moderators including intervention duration and frequency and mode of exercise may influence effects on muscle hypertrophy and suggest an “interference effect” [60,113,120].

### Limitations

The current systematic review and meta-analysis had several limitations that should be acknowledged. There was significant heterogeneity for several outcomes that may be due to diversity in the study methods, health statuses of participants, ages, and biological sexes. For two of the primary outcomes, muscle mass and CSA, there were few available studies, so it was not possible to examine those sources of potential heterogeneity. We acknowledge that measuring the main outcomes with less reliable methods, for example, using BIA and skinfolds, may have affected the validity and significance of the present findings. However, the results of the sensitivity analyses for these measurement methods confirmed the robustness of the results. In addition, exercise timing and volume are important moderators that may influence the effectiveness of exercise training interventions, and in a majority of included studies, these factors were not matched between intervention arms. Furthermore, it is important to note that the current meta-analysis excluded studies involving trained or athletic older adults to enhance the generalizability of our findings. This exclusion limits our ability to draw conclusions about the effects of combined training in trained or athletic populations. Future studies could focus on investigating the potential role of exercise intensity and volume on muscle mass in older adults, including trained/athletic and untrained individuals.

## 5. Conclusions

The current systematic review and meta-analysis provides evidence that CT is as effective as AT for decreasing body fat, and as effective as RT for increasing muscle mass in middle-aged and older adults. Since aging is associated with the loss of skeletal muscle and accumulation of fat mass, both of which are associated with detrimental changes in function and activities of daily living, CT can be recommended to optimize both aspects of body composition. However, it seems that the effects of CT are moderated by age and intervention duration, such that for some outcomes, middle-aged adults may be more likely to benefit from CT, and for some outcomes, longer-term interventions may be required.

## Figures and Tables

**Table 1:** Summary of participant demographic characteristics.

First Author, Year	Sample Size (Sex)	Health Status	Age (Years)	BMI (kg/m^2^)	Intervention Groups	Outcomes
Ahtiainen et al., 2009 [34]	26 (M)	Healthy	CT: 64 ± 3 AT: 58 ± 8 RT: 61 ± 5	CT: >28 AT: >28 RT: >28	CT AT RT	Weight, FP, LBM, CSA
Aminilari et al., 2017 [35]	37 (F)	Overweight and T2D	CT: 45–60 AT: 45–60 RT: 45–60	CT: 29.01 ± 2.57 AT: 30.03 ± 5.48 RT: 29.11 ± 1.92	CT AT RT	Weight, BMI, FP, MM
Balducci et al., 2010 [65]	42 (26 M, 16 F)	T2D and MetS	CT: 60.60 ± 9.30 AT: 64.30 ± 8.10	CT: 30.50 ± 0.90 AT: 29.40 ± 1.10	CT AT	Weight, BMI, WC, FP,
Bernard et al., 1999 [66]	36 (28 M, 8 F)	COPD	CT: 64.00 ± 7.00 AT: 67.00 ± 9.00	CT: 27.00 ± 5.00 AT: 25.00 ± 4.00	CT AT	CSA
Bouchla et al., 2011 [67]	20 (16 M, 4 F)	CHF	CT: 56.7 ± 7.2 AT: 50.5 ± 11.4	CT: 28.6 ± 4.4 AT: 28.1 ± 2.7	CT AT	LBM, FM
Boulé et al., 2013 [68]	62 (31 M, 31 F)	T2D	53.1 ± 6.9	33.3 ± 6.4	CT AT RT	Weight, WC
Burich et al., 2015 [69]	33 (6 M, 27 F)	Healthy	CT: 62.70 ± 5.10 AT: 63.60 ± 4.40	ND	CT AT	Weight, BF
Cadore et al., 2010 [36]	23 (M)	Healthy	CT: 66.80 ± 4.80 AT: 64.40 ± 3.50 RT: 64.00 ± 3.50	ND	CT AT RT	Weight, FM, BF
Campos et al., 2013 [37]	19 (F)	Healthy	CT1: 62.00 ± 2.50 CT2: 66.00 ± 3.50 AT: 63.60 ± 2.51 RT: 70.00 ± 6.27	CT1: 28.58 ± 7.63 CT2: 26.24 ± 4.90 AT: 27.40 ± 6.62 RT: 25.32 ± 4.24	CT1 CT2 AT RT	Weight, WC, FM, FP, MM
Chang et al., 2023 [70]	28 (3 M, 25 F)	Healthy and chronic disease (hypertension, T2D, cancer and MetS)	CT: 58.19 ± 8.16 AT: 59.59 ± 7.03	ND	CT AT	Weight, BMI, WC, FP, VF
Chen et al., 2017 [38]	45 (8 M, 37 F)	Sarcopenic obesity	CT: 68.50 ± 2.70 AT: 69.30 ± 3.00 RT: 68.90 ± 4.40	CT: 27.20 ± 2.90 AT: 26.80 ± 3.80 RT: 28.30 ± 4.40	CT AT RT	Weight, BMI, VF, MM, FP, FM
Choi et al., 2021 [71]	36 (F)	Overweight	CT: 73.19 ± 2.17 AT: 73.11 ± 2.11	CT: 25.11 ± 3.65 AT: 25.12 ± 2.11	CT AT	Weight, BMI, FP, LBM
Church et al., 2010 [39]	208 (78 M, 130 F)	T2D	CT: 55.40 ± 8.30 AT: 53.70 ± 9.10 RT: 56.90 ± 8.70	CT: 35.80 ± 6.20 AT: 34.70 ± 6.10 RT: 34.10 ± 5.40	CT AT RT	Weight, WC, FM, LBM
Cortez-Cooper et al., 2007 [72]	25 (6 M, 19 F)	Overweight	CT: 51.00 ± 1.00 RT: 52.00 ± 2.00	CT: 27.00 ± 1.10 RT: 26.80 ± 1.10	CT RT	Weight, BMI, FP, LBM
Cuff et al., 2003 [73]	19 (F)	T2D	CT: 63.40 ± 6.95 AT: 59.40 ± 5.70	CT: 33.30 ± 4.74 AT: 32.50 ± 4.20	CT AT	Weight, VF, CSA
Davidson et al., 2009 [40]	93 (37 M, 56 F)	Obesity	CT1: 67.10 ± 4.50 CT2: 66.50 ± 5.30 AT1: 68.80 ± 6.00 AT2: 69.10 ± 6.50 RT1: 67.40 ± 5.70 RT2: 67.60 ± 4.20	CT1: 31.10 ± 3.10 CT2: 29.50 ± 3.00 AT1: 29.90 ± 3.00 AT2: 29.20 ± 3.70 RT1: 30.10 ± 2.60 RT2: 30.00 ± 3.40	CT AT RT	Weight, BMI, WC, VF, MM, FM
Delaney et al., 2014 [74]	35 (26 M, 9 F)	Patients with intermittent claudication (overweight)	CT: 69.40 ± 9.60 AT: 73.40 ± 9.10	CT: 29.00 ± 5.60 AT: 27.00 ± 4.30	CT AT	MM
Delecluse et al., 2004 [75]	62 (M)	Overweight	CT1: 63.80 ± 4.80 CT2: 63.70 ± 6.00 AT: 64.50 ± 5.30	CT1: 27.10 ± 3.40 CT2: 26.80 ± 3.80 AT: 26.60 ± 2.80	CT1 CT2 AT	WC, FFM, FP
Do Amaral et al., 2021 [76]	28 (F)	Obesity	CT: 67.50 ± 3.40 RT: 66.10 ± 16.00	CT: 31.00 ± 7.90 RT: 30.00 ± 6.10	CT RT	Weight, BMI, WC
Feiereisen et al., 2007 [77]	45 (38 M, 7 F)	CHF	CT: 60.60 ± 5.60 AT: 59.40 ± 6.50 RT: 57.90 ± 5.80	ND	CT AT RT	MV
Gayda et al., 2009 [78]	16 (M)	CHD	55.00 ± 8.00	ND	CT AT	Weight, FP, LBM, MV
Gonzalo-Encabo et al., 2020 [79]	23 (F)	Overweight and obese postmenopausal	CT: 58 [56.5, 61.0] AT: 56.5 [55.0, 58.5]	CT: 32.5 [28.5, 37.5] AT: 32.3 [29.6, 35.5]	CT AT	Weight, FM, LBM
Hansen et al., 2011 [80]	47 (44 M, 3 F)	CAD	CT: 60.40 ± 8.90 AT: 58.90 ± 7.20	CT: 26.60 ± 3.10 AT: 27.00 ± 3.20	CT AT	FM, LBM
Ho et al., 2012 [41]	48 (6 M, 42 F)	Overweight obese	CT: 53.00 ± 1.30 AT: 55.00 ± 1.20 RT: 52.00 ± 1.10	CT: 33.30 ± 1.20 AT: 32.70 ± 1.30 RT: 33.00 ± 1.30	CT AT RT	Weight, BMI, WC, FM, FP
Irving et al., 2015 [42]	30 (15 M, 15 F)	Healthy	CT: 71.00 ± 6.32 AT: 70.00 ± 3.31 RT: 70.00 ± 3.31	CT: 28.00 ± 3.16 AT: 28.00 ± 3.31 RT: 27.00 ± 3.31	CT AT RT	FP, FM, LBM, CSA
Izquterdo et al., 2004 [43]	31 (M)	Healthy	CT: 66.40 ± 4.50 AT: 68.20 ± 1.70 RT: 64.80 ± 2.60	CT: 25.20 ± 8.70 AT: 27.60 ± 2.70 RT: 29.60 ± 4.10	CT AT RT	Weight, BMI, FP, FFM, CSA
Jorge et al., 2011 [81]	36 (14 M, 22 F)	T2D	CT: 57.90 ± 8.06 AT: 52.09 ± 8.71 RT: 54.10 ± 8.93	CT: 31.23 ± 3.88 AT: 29.29 ± 2.19 RT: 30.89 ± 4.09	CT AT RT	BMI
Kadoglou et al., 2013 [44]	66 (18 M & 48 F)	T2D	CT: 57.90 ± 6.50 AT: 58.30 ± 5.40 RT: 56.10 ± 5.30	CT: 31.91 ± 2.93 AT: 31.55 ± 3.11 RT: 32.89 ± 3.26	CT AT RT	BMI, FP
Karavirta et al., 2011 [45]	30 (M)	Healthy	CT: 56.00 ± 7.00 AT: 54.00 ± 8.00 RT: 56.00 ± 6.00	CT: 26.20 ± 3.20 AT: 25.50 ± 3.40 RT: 26.40 ± 2.90	CT AT RT	CSA
Kim et al., 2018 [82]	25 (3 M, 22 F)	Healthy	73.20 ± 4.90	CT: 24.94 ± 1.63 RT: 25.75 ± 2.60	CT RT	Weight, BMI, LBM, FP, WC
Kobayashi et al., 2023 [46]	186 (112 M, 74 F)	T2D	CT: 59 (52–68) AT: 59 (54–65) RT: 60 (53–66)	CT: 23.80 (22.9–24.7) AT: 23.60 (22.2–24.7) RT: 23.50 (22.6–24.4)	CT AT RT	LBM, FM
Lee et al., 2015 [83]	19 (F)	Healthy	CT: 68.38 ± 2.93 AT: 67.64 ± 2.82	CT: 24.23 ± 1.33 AT: 24.09 ± 1.28	CT AT	Weight, BMI, FM, LBM, FP
Lima et al., 2017 [84]	30 (5 M, 25 F)	Hypertension	CT: 67.80 ± 5.20 AT: 67.80 ± 4.30	CT: 28.00 ± 3.20 AT: 28.90 ± 3.50	CT AT	BMI, LBM, FM, WC
Marzolini et al., 2018 [85]	68 (44 M, 24 F)	Chronic stroke with motor impairments	CT: 61.70 ± 10.00 AT: 65.60 ± 13.20	CT: 27.78 ± 4.30 AT: 25.32 ± 5.10	CT AT	BMI, LBM, FP
Marzolini et al., 2008 [86]	53 (47 M, 6 F)	CAD	CT1: 62.70 ± 11.45 CT2: 60.90 ± 10.02 AT: 57.90 ± 10.40	CT1: 25.70 ± 3.01 CT2: 27.60 ± 3.61 AT: 26.80 ± 3.48	CT AT	LBM, FP
Moreno-Cabañas et al., 2021 [87]	66 (48 M, 18 F)	MetS	56.00 ± 7.00	CT: 32.70 ± 5.20 AT: 32.00 ± 4.10	CT AT	Weight, BMI, FM, FFM, WC
Pedralli et al., 2020 [88]	37 (20 M, 17 F)	Prehypertension or hypertension	CT: 53.80 ± 9.10 AT: 50.90 ± 14.20 RT: 55.10 ± 6.90	CT: 27.90 ± 5.50 AT: 29.80 ± 4.10 RT: 28.50 ± 6.01	CT AT RT	Weight, BMI, WC
Piralaiy et al., 2021 [47]	33 (M)	T2D neuropathy	55.24 ± 8.11	CT: 30.36 ± 3.02 AT: 30.39 ± 3.36 RT: 28.79 ± 2.59	CT AT RT	Weight, BMI, FP
Rossi et al., 2016 [89]	52 (F)	Hyper-triacylglycerolemic postmenopause	61.0 ± 6.3	CT: 28.3 ± 2.7 AT: 28.4 ± 2.9	CT AT	Weight, BMI, FM, FP, FFM
Rossi et al., 2018 [90]	29 (F)	Postmenopause	CT: 61.40 ± 5.00 AT: 61.80 ± 7.9	CT: 30.50 ± 4.30 AT: 28.40 ± 2.90	CT AT	FM, LBM
Ruangthai et al., 2019 [48]	42 (6 M, 36 F)	Hypertension	CT: 67.30 ± 5.90 AT: 65.60 ± 4.50 RT: 68.00 ± 7.40	CT: 24.10 ± 2.00 AT: 23.80 ± 2.40 RT: 22.60 ± 2.60	CT AT RT	Weight, BMI, FM, FP, MM
Schroeder et al., 2019 [49]	52 (21 M, 31 F)	Hypertension, overweight/obesity	CT: 58.00 ± 7.00 AT: 58.00 ± 7.00 RT: 57.00 ± 9.00	CT: 31.90 ± 5.50 AT: 32.50 ± 5.90 RT: 33.10 ± 5.90	CT AT RT	Weight, BMI, WC, LBM, FM (kg), FP
Scott et al., 2021 [50]	67 (24 M, 43 F)	Lung cancer survivors with poor cardiorespiratory fitness	CT: 63.00 ± 11.00 AT: 64.00 ± 9.00 RT: 64.00 ± 9.00	CT: 27.00 ± 4.00 AT: 26.00 ± 6.00 RT: 27.00 ± 6.00	CT AT RT	Weight, LBM, FP
Sénéchal et al., 2013 [91]	171 (62 M, 109 F)	T2D	CT: 55.90 ± 7.20 AT: 55.10 ± 8.30 RT: 58.10 ± 8.30	CT: 34.70 ± 5.90 AT: 34.00 ± 5.60 RT: 33.90 ± 5.40	CT AT RT	Weight, BMI, WC, FP, FM, LBM
Seo et al., 2010 [92]	15 (F)	Healthy postmenopausal	CT: 54.00 ± 3.60 AT: 55.00 ± 4.80	CT: 24.00 ± 1.90 AT: 27.40 ± 3.40	CT AT	Weight, BMI, WC, FP
Sigal et al., 2007 [93]	188 (119 M, 69 F)	T2D	CT: 53.50 ± 7.30 AT: 53.90 ± 6.60 RT: 54.70 ± 7.50	CT: 35.00 ± 9.60 AT: 35.60 ± 10.10 RT: 34.10 ± 9.60	CT AT RT	Weight, BMI, WC, LBM, FM, FP, VF, CSA
Sillanpää et al., 2010 [94]	70 (F)	Healthy	CT: 51.00 ± 7.00 AT: 53.00 ± 8.00 RT: 52.00 ± 8.00	CT: 25.00 ± 3.10 AT: 25.10 ± 2.60 RT: 24.70 ± 3.20	CT AT RT	BMI, FP, LBM
Sillanpää et al., 2008 [95]	42 (M)	Healthy	CT: 56.30 ± 6.80 AT: 54.10 ± 7.70 RT: 54.60 ± 6.10	CT: 24.70 ± 3.00 AT: 23.70 ± 2.00 RT: 25.30 ± 1.90	CT AT RT	Weight, BMI, WC, LBM, FP
Sparks et al., 2013 [96]	42 (21 M, 21 F)	T2D	CT: 54.10 ± 6.20 AT: 54.20 ± 6.00 RT: 60.40 ± 7.30	CT: 37.10 ± 6.80 AT: 33.40 ± 5.80 RT: 33.90 ± 5.20	CT AT RT	BMI, FP, FFM
Swift et al., 2012 [97]	167 (69 M, 98 F)	T2D	CT: 56.70 ± 7.80 AT: 55.80 ± 7.90 RT: 58.70 ± 8.00	CT: 35.00 ± 6.20 AT: 33.90 ± 5.70 RT: 34.10 ± 5.40	CT AT RT	Weight, WC, FP, FM, LBM
Theodorou et al., 2016 [98]	41 (M)	CAD	CT: 64.00 ± 6.00 AT: 61.00 ± 7.00 RT: 62.00 ± 8.00	CT: 29.80 ± 0.90 AT: 31.10 ± 0.70 RT: 31.60 ± 1.40	CT AT RT	Weight, BMI, FP
Timmons et al., 2018 [99]	63 (37 M, 26 F)	Healthy	CT: 69.20 ± 2.70 AT: 69.20 ± 3.10 RT: 69.60 ± 4.90	CT: 27.50 ± 3.70 AT: 24.90 ± 4.0 RT: 26.90 ± 3.60	CT AT RT	Weight, FP, FM, LBM
Zhou et al., 2022 [100]	66 (21 M, 45 F)	MetS	CT1: 82.82 ± 3.81 CT2: 78.41 ± 6.12 AT: 79.46 ± 4.82 RT: 78.88 ± 5.40	CT1: 24.54 ± 3.97 CT2: 24.82 ± 2.92 AT: 24.05 ± 3.00 RT: 26.09 ± 3.05	CT1 CT2 AT RT	BMI, WC

Abbreviations: AT: aerobic training; CAD: coronary artery disease; CHF: chronic heart failure; CKD: chronic kidney disease; COPD: chronic obstructive pulmonary disease; CHD: chronic heart disease; CT: concurrent training; RT: resistance training; CSA: muscle cross-sectional area; DEXA: dual-energy X-ray absorptiometry; F: female; FM: fat mass; FFM: fat free mass; HR_max/peak_: maximal/peak heart rate; ND: not described; MM: muscle mass; MetS: metabolic syndrome; MV: muscle volume; LBM: lean body mass; reps: repetitions; BRPE: Borg rating of perceived exertion; VF: visceral fat; WC: waist circumference; 1RM: one-repetition maximum.

**Table 2:** Summary of exercise training interventions.

First Author, Year	Concurrent Training (CT)	Same Session or Separate	Aerobic Training (AT)	Resistance Training (RT)	Supervised /Non-Supervised	Frequency (Times /Week)	Intervention Duration
Ahtiainen et al., 2009 [34]	AT: 30–90 min, under the level of aerobic threshold to a steady pace under the aerobic threshold RT: 3 sets, 15–30 reps, 40–90% 1RM, 1–3 min rests between sets, including leg press, knee extension and flexion, bench press, triceps pushdown, lateral pull-down, sit-up, and elbow flexion	Separate	30–90 min, under the level of aerobic threshold to a steady pace under the aerobic threshold, cycling	3 sets, 15–30 reps, 40–90% 1RM, 1–3 min rests between sets, including leg press, knee extension and flexion, bench press, triceps pushdown, lateral pull-down, sit-up, elbow flexion	Supervised	CT: 4 AT: 2 RT: 2	21 weeks
Aminilari et al., 2017 [35]	AT: 25 min, 50–55% HR_max_ RT: 3 sets, 8 reps, 50–55% 1RM, including leg extension, prone leg curl, abdominal crunch, biceps, triceps, and seated calf	Same	25 min, 50–55% HR_max_, cycling	3 sets, 8 reps, 50–55% 1RM, including leg extension, prone leg curl, abdominal crunch, biceps, triceps, and seated calf	ND	3	12 weeks
Balducci et al., 2010 [65]	AT: 40 min, 70–80% VO_2max_ RT: 20 min, 80% 1RM, including chest press, lateral pull-down, leg press, and trunk flexion	Same	60 min, 70–80% VO_2max_, treadmill/cycling	–	Supervised	2	12 months
Bernard et al., 1999 [66]	AT: 30 min, 80% work rate achieved during the baseline incremental exercise test RT: 2–3 sets, 8–10 reps, 60–80% 1RM, including seated press, elbow flexion, shoulder adduction, leg press, and bilateral knee extension	Same	30 min, 80% work rate achieved during the baseline incremental exercise test, cycling	–	Supervised	3	12 weeks
Bouchla et al., 2011 [67]	AT: 20 min, interval training (30-s exercise, 60-s rest), 50% of the workload peak RT: 20 min, 3 sets, 10–12 reps, 30-s rest between the sets; intensity for quadriceps was 55–65% of 2RM, intensity for the hamstrings was that of quadriceps minus 0.5–1 kg, intensity for the upper extremities exercises was set at 10RM including leg extension, leg curls, arm curls, and lateral arm abduction	Same	40 min, interval (30 s exercise, 60 s rest), 50% of the workload peak, cycling	40 min, 3 sets, 10–12 reps, 30-s rest between the sets; intensity for quadriceps was 55–65% of 2RM, intensity for the hamstrings was that of quadriceps minus 0.5–1 kg, intensity for the upper extremities exercises was set at 10RM including leg extension, leg curls, arm curls, and lateral arm abduction	Supervised	3	12 weeks
Boulé et al., 2013 [68]	AT: 45 min, 75% HR_max_ RT: 2–3 sets, 7–9 reps, maximum weight including 7 exercises on weight machines	Same	45 min, 75% HR_max_, aerobic training progressed	2–3 sets, 7–9 reps, maximum weight, including 7 exercises on weight machines	Supervised	3	22 weeks
Burich et al., 2015 [69]	AT: 20 min, 65% HRR RT: 3 sets, 10–15 reps, including leg press, calf extension, and knee extension	Same	40 min, 65% HRR, cycling	–	Supervised	3	12 weeks
Cadore et al., 2010 [36]	AT: 20–30 min, 80–100% heart rate at VT_2_ RT: 2–3 sets, 6–20 reps, until failure including leg press, knee extension, leg curl, bench press, lat pull-down, seated row, triceps curl, biceps curl, and abdominal exercises	Same	20–30 min, 80–100% heart rate at VT_2_, cycling	2–3 sets, 6–20 reps, until failure including leg press, knee extension, leg curl, bench press, lat pull-down, seated row, triceps curl, biceps curl, and abdominal exercises	Supervised	3	12 weeks
Campos et al., 2013 [37]	AT: 20–30 min, 65–85% MHR, treadmill RT: 3 sets, 4–20 reps, including chest press, behind the neck lat pull-down, knee extension, knee flexion, biceps pulley, triceps pulley, and leg press 3 sets, 30 reps, curl abdominal on the ground	Same	20–30 min at 65–85% MHR, treadmill	3 sets, 4–20 reps, including chest press, behind the neck lat pull-down, knee extension, knee flexion, biceps pulley, triceps pulley, and leg press 3 sets, 30 reps, curl abdominal on the ground	ND	3	12 weeks
Chang et al., 2023 [70]	AT: 30 min, implemented core muscle training including side swings, rocking horses, marches, and jumping jacks RT: 3–5 sets, 8–12 reps, 30 s rest between sets, 10 min including free weights or a 25-pound theraband including leg press, chest press, lateral pull-downs, shoulder press, arm curls, and triceps extensions	Same	AT: 30 min, implemented core muscle training including side swings, rocking horses, marches, and jumping jacks	–	Supervised and non-supervised	3	12 weeks
Chen et al., 2017 [38]	AT: 40–45 min, combination of dance steps including dance steps such as stepping on the spot, knee lifts, high knee running, rowing arm swings, arm swings, twist steps, arm raises, squats, V steps, mambo steps, diamond steps, and point step jumps RT: 3 sets, 8–12 reps, 60–70% 1RM including shoulder presses, bicep curls, triceps curls, bench presses, deadlifts, leg swings, squats, standing rows, unilateral rows, and split front squats	Separate	40–45 min, combination dance steps including stepping on the spot, knee lifts, high knee running, rowing arm swings, arm swings, twist steps, arm raises, squats, V steps, mambo steps, diamond steps, and point step jumps	3 sets, 8–12 reps, 60–70% 1RM, including shoulder presses, bicep curls, triceps curls, bench presses, deadlifts, leg swings, squats, standing rows, unilateral rows, and split front squats	ND	2	8 weeks
Choi et al., 2021 [71]	AT: 20 min, 60–70% HR_max/peak_ RT: 3 sets, 15 reps, 60–70% 1RM including squat, wide squat, tubing band squat, tubing band wide squat, single leg-link exercise, double leg-link exercise, and tubing band leg-link exercise	Same	50 min, 60–70% HR_max/peak_, walking/jogging	–	ND	3	12 weeks
Church et al., 2010 [39]	AT: 110 min per week, 50–80% VO_2max/peak_ RT: 1 set, 10–12 reps, including bench press, seated row, shoulder press, pull-down, leg press, extension, flexion, abdominal crunches, and back extensions	ND	140 min per week, 50–80% VO_2max/peak_, treadmill	2–3 sets, 10–12 reps, including bench press, seated row, shoulder press, pull-down, leg press, extension, flexion, abdominal crunches, and back extensions; one set with	Supervised	3	9 months
Cortez-Cooper et al., 2007 [72]	AT: 30–45 min, 60–75% HRR, 2 days/week, walking or cycling RT: 1 set, 8–12 reps, 70% 1RM, including seated chest press, horizontal leg press, shoulder press, abdominal crunches, seated hamstring curls, seated row, seated calf raises, low back extension, triceps curls, and bicep dumbbell curls, 2 days/week	Separate	–	1 set, 8–12 reps, 70% 1RM, including seated chest press, horizontal leg press, shoulder press, abdominal crunches, seated hamstring curls, seated row, seated calf raises, low back extension, triceps curls, and bicep dumbbell curls	Supervised and non-supervised	CT: 4 RT: 3	13 weeks
Cuff et al., 2003 [73]	AT: 65–75% HRR, treadmill, bicycle, stepper, elliptical trainers, and rowing machines RT: 2 sets, 12 reps, began with light loads and there after progressed including leg press, leg curl, hip extension, chest press, and latissimus pull-down	Same	75 min, 65–75% HRR, treadmill, bicycle, stepper, elliptical trainer, and rowing machines	–	ND	3	16 weeks
Davidson et al., 2009 [40]	AT: 30 min, 60–75% VO_2max/peak_ RT: 1 set with reps to volitional fatigue including chest press, shoulder raise, shoulder flexion, leg extension, leg flexion, triceps extension, biceps curl, abdominal crunches, and modified push-ups	Same	30 min, 60–75% VO_2max/peak_, walking	1 set with reps to volitional fatigue including chest press, shoulder raise, shoulder flexion, leg extension, leg flexion, triceps extension, biceps curl, abdominal crunches, and modified push-ups	Supervised	CT: 3 AT: 5 RT: 3	6 months
Delaney et al., 2014 [74]	AT: 60 min, walk until claudication pain became unbearable, then rested and repeated the cycle Initial treadmill speed was determined by distance covered in the baseline 6 min walking test If the participant did not experience symptoms within 10 min of walking, the pace or gradient of walking was increased by 10% RT: 3 sets, 8–12 reps, including hamstring curls, seated calf press, knee extension, and hip abduction/adduction	Same	60 min, walk until claudication pain became unbearable, then rested and repeated the cycle Initial treadmill speed was determined by distance covered in the baseline 6-min walking test; if the participant did not experience symptoms within 10 min of walking, the pace or gradient of walking was increased by 10%	–	Supervised	2	12 weeks
Delecluse et al., 2004 [75]	AT: 12–20 min, 60–80% HRR RT1: 2 sets, 8–20 reps, moderate to high resistance load, including leg press, leg extension, leg curl, adductor, abductor, vertical row, chest press, arm curl, shoulder press, and abdomen RT2: 2 sets, 30 reps, low resistance load during including leg press, leg extension, leg curl, adductor, abductor, vertical row, chest press, arm curl, shoulder press, and abdomen	Same	36–70 min, 60–80% HRR, cycling/walking	–	Supervised	2–3	20 weeks
Do Amaral et al., 2021 [76]	AT: 15–30 min, walking at 11–13 RPE RT:1–2 sets, 10–20 reps, 15–17 RPE including bodyweight squat, push-up on the wall or counter, deadlift with a stich or rubber band, shoulder press or shoulder abduction with rubber band, and abdominal crunch on the floor or chair	Same	–	2–4 sets, 10–20 reps, 15–17 RPE, 20–50 min, including bodyweight squat, push-up on the wall or counter, deadlift with a stich or rubber band, shoulder press or shoulder abduction with rubber band, and abdominal crunch on the floor or chair	Supervised	2	12 weeks
Feiereisen et al., 2007 [77]	AT: 20 min, 60–75% VO_2max/peak_ RT: 4 sets, 10 reps, 60–70% 1RM, including pull-down, reverse butterfly, rowing, knee extension, and knee flexion	Same	40 min, 60–75% VO_2max/peak_, cycling and treadmill	4 sets, 10 reps, 60–70% 1RM, including pull-down, reverse butterfly, rowing, arm abduction, knee extension, knee flexion, leg press, calf raises, and trunk flexion and trunk extension	ND	3	3 months
Gayda et al., 2009 [78]	AT: 60 min, ventilatory threshold power RT: 3 sets, 10 reps, 40% MVC including quadriceps leg extension and lateral pull-down	Separate	60 min, ventilatory threshold power, cycling/calisthenics movements, stretching, and respiratory exercises	–	ND	CT: 6 AT: 3	7 weeks
Gonzalo-Encabo et al., 2020 [79]	AT: 10–20 min, 55–75% of HRR, treadmill, cycle ergometer, and elliptical machine RT: 2–3 sets of 8–12 reps, 65% 1RM, 60–90 s rest, 40 min/session, including chest press, seated row, leg press, leg extension, biceps curl, and triceps extension	Same	10–60 min, 55–75% of HRR, treadmill, cycle ergometer, and elliptical machine	–	Supervised	3	12 weeks
Hansen et al., 2011 [80]	AT: 40 min, 65% VO_2max/peak_ RT: 2 sets, 12–20 reps, 65% 1RM, including leg extension leg press	Same	40 min, 65% VO_2max/peak_, cycling/walking/arm cranking	–	Supervised	3	6 weeks
Ho et al., 2012 [41]	AT: 15 min, treadmill walking RT: 2 sets, 15 min, including leg press, leg curl, leg extension, bench press, and rear deltoid row, with each set completed in ~30 s with 1 min rest	Same	30 min, 60% HRR, treadmill walking	4 sets, 8–12 reps, 10RM, 30 min, including leg press, leg curl, leg extension, bench press, and rear deltoid row, with each set completed in ~30 s with 1 min rest	Supervised and non-supervised	5	12 weeks
Irving et al., 2015 [42]	AT: 30 min, 65% VO_2max/peak_ RT: 4 sets, 8–10 reps, multiple muscle groups	Same	60 min, 65% VO_2max/peak_, cycling	4 sets, 8–10 reps, multiple muscle groups	Supervised	CT: 5 AT: 5 RT: 4	8 weeks
Izquterdo et al., 2004 [43]	AT: 30–40 min, 70–90% HR_max_, cycling at a constant rate of 60 rmp RT: Week 1–8: 3–4 sets, 10–15 reps, 50–70% 1RM, 45–60 min Week 8–16: 3–5 sets, 5–6 reps, 70–80% 1RM, 45–60 min Week 1–16: exercises included bilateral leg press and bilateral knee extension exercises, the bench press, chest press, lateral pull-down, and/or shoulder press, abdominal crunch and/or rotary torso and/or another exercise for the trunk extensors, and the standing leg curl and/or adductor–abductor exercises Week 8–6: 3–4 sets, 6–8 reps including a part (20%) of the leg extensor and bench press sets with the loads ranging from 30% to 50% and 30% to 40% of the maximum, respectively	Separate	30–40 min, 70–90% HR_max_, cycling at a constant rate of 60 RPM	Week 1–8: 3–4 sets, 10–15 reps, 50–70% 1RM, 45–60 min Week 8–16: 3–5 sets, 5–6 reps, 70–80% 1RM, 45–60 min Week 1–16: exercises included bilateral leg press and bilateral knee extension exercises, the bench press, chest press, lateral pull-down, and/or shoulder press, abdominal crunch and/or rotary torso and/or another exercises for the trunk extensors, and the standing leg curl and/or adductor-abductor exercises Week 8–6: 3–4 sets, 6–8 reps, including a part (20%) of the leg extensor and bench press sets with the loads ranging from 30% to 50% and 30% to 40% of the maximum, respectively	Supervised	CT: 1 AT: 1 RT: 2	16 weeks
Jorge et al., 2011 [81]	AT: 30 min, cycling at the heart rate corresponding to the lactate threshold RT: 30 min, including leg press, bench press, lat pull-down, seated rowing, shoulder press, abdominal curls, and knee curls	Same	60 min, cycling at the heart rate corresponding to the lactate threshold	60 min, including leg press, bench press, lat pull-down, seated rowing, shoulder press, abdominal curls, and knee curls	Supervised	3	12 weeks
Kadoglou et al., 2013 [44]	Half the volume of AT: 60 min, 60–75% HR_max/peak_, walking or running, and cycling or calisthenics RT: 2–3 sets, 8–10 reps, 60–80% 1RM, including seated leg press, knee extension, knee flexion, chest press, lat pull-down, overhead press, biceps curl, and triceps extension)	Same and Separate	60 min, 60–75% HR_max/peak_, walking or running, cycling or calisthenics	2–3 sets, 8–10 reps, 60–80% 1RM, including seated leg press, knee extension, knee flexion, chest press, lat pull-down, overhead press, biceps curl, and triceps extension	Supervised	4	6 months
Karavirta et al., 2011 [45]	AT: Weeks 1–7: 30 min below the level of the aerobic threshold Weeks 5–7: intensity was above the aerobic threshold by a 10 min interval in the middle of the sessions Weeks 8–14: 45 min included a 10 min interval between the aerobic-anaerobic thresholds and a 5 min interval above the anaerobic threshold The other weekly training session involved 60 min of cycling below the aerobic threshold Weeks 15–21: one of weekly sessions lasted for 60 min, included 2 10 min intervals between the aerobic-anaerobic thresholds, 2 5 min intervals above the anaerobic threshold and 30 min below the aerobic threshold; the other weekly session included 90 min of cycling at a steady pace below the aerobic threshold RT:1st cycle: 3 sets, 12–20 reps, 40–60% 1RM RT 2nd cycle: 2–4 sets, 5–12 reps, 60–80% 1RM RT 3rd cycle: 2–4 sets, 5–8 reps, 70–85% 1RM Including leg press, knee extension, leg curl, seated calf raise, hip abduction or adduction, bench press, biceps curl, triceps push-down, lateral pull-down, abdominal crunch, and seated back extension. In addition, ~20% of the leg press, knee extension and bench press exercises were performed with light loads of 40–50% of 1RM and 5–8 reps	Separate	Weeks 1–7: 30 min, below the level of the aerobic threshold. Weeks 5–7: intensity was above the aerobic threshold by a 10 min interval in the middle of the sessions Weeks 8–14: 45 min included a 10 min interval between the aerobic-anaerobic thresholds and a 5 min interval above the anaerobic threshold; the other weekly training session involved 60 min of cycling below the aerobic threshold Weeks 15–21: one of weekly sessions lasted for 60 min, included two 10 min intervals between the aerobic-anaerobic thresholds, two 5 min intervals above the anaerobic threshold and 30 min below the aerobic threshold; the other weekly session included 90 min of cycling at a steady pace below the aerobic threshold	1st cycle: 3 sets, 12–20 reps, 40–60% 1RM 2nd cycle: 2–4 sets, 5–12 reps, 60–80% 1RM 3rd cycle: 2–4 sets, 5–8 reps, 70–85% 1RM Including leg press, knee extension, leg curl, seated calf raise, hip abduction or adduction, bench press, biceps curl, triceps push-down, lateral pull-down, abdominal crunch, and seated back extension In addition, 5–8 reps, 40–50% 1RM, ~20% of the leg press, knee extension and bench press exercises were performed with light loads	Supervised	AT and RT: 2 CT: 4	21 weeks
Kim et al., 2018 [82]	AT: 2 sets, 6–8 RPE, 20 min, including sky-walk and cross-country RT: 1–3 sets, 12–15 reps, 6–8 RPE, 50 min, including pull weight, chair pull, leg extension	Same	–	1–3 sets, 12–15 reps, 6–8 RPE, 70 min, including pull weight, chair pull, leg extension	Supervised	3	6 weeks
Kobayashi et al., 2023 [46]	AT: 50–80% peak MET, AT was standardized to body weight at 41.8 kJ (kcal) kg body weight^−1^ week^−1^ on a treadmill, an elliptical, or a stationary bike RT: 1 set including bench press, seated row, shoulder press, pull-down, leg press, extension and flexion, and abdominal crunches, and back extensions, 8–12 reps	Same	50–80% peak MET, AT was standardized to body weight at 50.2 kJ (kcal) kg body weight^−1^ week^−1^ on a treadmill, an elliptical or a stationary bike	2 sets (including bench press, seated row, shoulder press, and pull-down), 3 sets (including leg press, extension and flexion), and 2 sets (including abdominal crunches and back extensions), 8–12 reps	Supervised	3	9 months
Lee et al., 2015 [83]	AT: 40 min, 40–70% HRR RT: 2 sets, 15–20 reps, 10–13 RPE including ankle dorsiflexion, ankle plantar flexion, ankle eversion, ankle inversion, leg press, knee extension, knee flexion, hip extension, hip flexion, hip abduction, hip adduction, crunches, and hyperextensions	Separate	40 min, 40–70% HRR, treadmill	–	ND	5	8 weeks
Lima et al., 2017 [84]	AT: 20–30 min, treadmill ergometer, intensity was based on the physical conditioning of each participant RT: 1–2 sets, 15 reps upper limbs, 20 reps trunk and lower limbs, 50–60% 1RM, including leg press 45°, bench press, extensor bench, handle front, flexor bench-seated, upright row, plantar flexion, seated row, and abdominals	Same	20–30 min, treadmill ergometer, intensity was based on the physical conditioning of each participant	–	Supervised	3	10 weeks
Marzolini et al., 2018 [85]	AT: 20–60 min, 60–80% HRR RT: 1–2 sets, 10–15 reps, 50–70% 1RM including lunge, squat, abdominal curl-up, heel raise, bicep curl, supine triceps extension, affected-side hip flexion/extension, affected-side ankle dorsiflexion, and single-limb knee extension and flexion	Separate	20–60 min, 60–80% HRR, cycling/walking	–	Supervised and non- supervised	5	6 months
Marzolini et al., 2008 [86]	AT: 30–60 min, 60–80% VO_2max/peak_ RT: 1–3 set, 10–15 reps, 60–75% 1RM, including bent over dumbbell row, half squat, alternating right and left arm bicep curl, heel raises, standing lateral raise, leg curl, supine lateral raise, curl up abdominal exercise, tricep extension, and four-point alternate arm and leg lift	Separate	30–60 min, 60–80% VO_2max/peak_, cycling/jogging	–	Supervised and non- supervised	5	24 weeks
Moreno-Cabañas et al., 2021 [87]	AT: 4 × 4 min, 90% HR_max/peak_, 3 min recovery 70% HR_max/peak_ RT: 3 sets, 10 reps, 20–30% 1RM, including squat, lunge, and dead lift	Same	5 × 4 min, 90% HR_max/peak_; 3 min recovery 70% HR_max/peak_, cycling	–	Supervised	3	16 weeks
Pedralli et al., 2020 [88]	AT: 20 min, 50–75% HRR RT: 2 sets, 12 reps, 20 min, 60–80% 1RM, including leg press, bench press, knee extension, biceps direct threading, knee flexion, and low row	Same	40 min, 50–70% HRR or 11–14 RPE, including cycle ergometer	4 sets, 8–12 reps, ~40 min with 60–80% 1RM including leg press, bench press, knee extension, biceps direct threading, knee flexion, and low row	ND	2	8 weeks
Piralaiy et al., 2021 [47]	AT: 25–45 min, 70% MHR RT: 1 set, each exercise at the maximum weight that could be lifted 8–12 times including bench press, seated row, shoulder press, pull-down, leg press/extension/flexion, abdominal crunches, and back extensions	Separate	25–45 min, 70% MHR, full aerobic-training program	2–3 sets, each exercise at the maximum weight that could be lifted 8–12 times including bench press, seated row, shoulder press, pull-down, leg press/extension/flexion, abdominal crunches, and back extensions	Supervised	CT: 5 AT: 3 RT: 3	12 weeks
Rossi et al., 2016 [89]	AT: 30 min, 100% critical velocity RT: 3 sets, 8–15 reps, 60 s between sets, 30 min including 45° leg press, leg extension, leg curl, bench press, seated row, arm curl, triceps extension, side elevation with dumbbells, and abdominal exercises	Same	50 min, 100% critical velocity	–	ND	ND	16 weeks
Rossi et al., 2018 [90]	AT: 30 min, 100% of critical velocity RT: 3–4 sets, 8–15 reps at ~65–80% of maximum repetitions, 60–90 s between sets, 27 min including 45° leg press, leg extension, leg curl, bench press, seated row, arm curl, triceps extension, side elevation with dumbbells, and abdominal exercises	Same	52 min, 100% of critical velocity	–	ND	ND	16 weeks
Ruangthai et al., 2019 [48]	AT: 20 min, 50–70% HR_max,_ including walk with arms up, heel hit behind, tiptoe, arms adduction/abduction, and knee lift RT: 2 sets, 8–12 reps, 50–80% 1RM, 60 min including squat, legs raise, knee extension, unilateral knee flexion, leg adduction/abduction, leg kick back, shoulder press, bench press, bicep curl, triceps dip, lateral flexion, sit-up, and back extension	Same	60 min, 50–70% HR_max,_ including walk with arms up, heel hit behind, tiptoe, arms adduction/abduction, and knee lift	3 sets, 10–15 reps, 50–80% 1RM, 60 min, including squat, legs raise, knee extension, unilateral knee flexion, leg adduction/abduction, leg kick back, shoulder press, bench press, bicep curl, triceps dip, lateral flexion, sit-up, and back extension	Supervised and non-supervised	3	24 weeks
Schroeder et al., 2019 [49]	AT: 30 min, 40–80% HRR RT: 2 sets, 10–20 reps, maximal load including chest press, pull-down, lower-back extension, abdominal crunch, torso rotation, leg press, leg curl, and hip abduction	Same	60 min, 40–80% HRR, treadmill/cycling	2–3 sets, 10–20 reps, maximal load including chest press, shoulder press, pull-down, lower-back extension, abdominal crunch, torso rotation, biceps curl, triceps extension, leg press, quadriceps extension, leg curl, and hip abduction	Supervised	3	8 weeks
Scott et al., 2021 [50]	Weeks 1–8: 3 AT plus RT sessions per week Weeks 9–16: 2 AT plus RT and 1 interval AT sessions per week CT followed AT and RT dosing schedules for a total of 30–90 min per session; within each session, AT was performed first followed by RT	Same	20–60 min, 55->95% of VO_2peak_, interval sessions consisted of 60–120 s at VO_2peak_ followed by 120–180 s of active recovery for 4–10 intervals per session consisted of stationary cycle ergometry	2–3 sets, 6–18 reps, 50–85% including chest press, seated row, lateral pull-down, pec deck, bicep curl, triceps extension, push-up, leg press, leg curl, leg extension, hip abduction, hip adduction, step up, and sit-to-stand	Supervised	3	16 weeks
Sénéchal et al., 2013 [91]	AT: an aerobic exercise dose of 10 kcal/kg body weight per week RT:1 set of 10–12 reps including bench press, seated row, shoulder press, lat pull-down, leg press, leg extension, and leg curl, abdominal crunches, and back extensions	Separate	50–80% VO_2peak_	2 sets including bench press, seated row, shoulder press, and lat pull-down, 3 sets including leg press, leg extension, and leg curl, and 2 sets of abdominal crunches and back extensions, 10–12 reps	Supervised	AT: 3–5 RT: 3	9 months
Seo et al., 2010 [92]	AT: 60–80% HRR, walked and aerobic exercise RT: 3 sets at 50–70% of 1RM, 30–40 min including seated chest press, lat pull-down, seated leg extension, leg curl, biceps curl, seated triceps extension, shoulder press, and sit-up	ND	60–80% HRR, walked and aerobic exercise	–	Supervised	3	12 weeks
Sigal et al., 2007 [93]	AT: 15–45 min, 60–75% HR_max_ treadmill/bicycle ergometers RT: 2–3 sets, the maximum weight that could be lifted 7–9 times	Same	15–45 min, 60–75% HR_max_ treadmill/bicycle ergometers	2–3 sets, the maximum weight that could be lifted 7–9 times	Supervised and non-supervised	3	6 months
Sillanpää et al., 2010 [94]	AT plus RT	ND	Week 1–5: intensity was under the level of aerobic threshold and lasted 30 min Weeks 5–7: intensity was between the aerobic and anaerobic thresholds to accustom to higher intensities Weeks 8–14: intensity was under the aerobic threshold (60 min) and every second session included 45 min cycling with intensities varying from under the aerobic threshold to over the anaerobic threshold; during the last training weeks, every other training session included 75–90 min of cycling at a steady pace under the aerobic threshold and every other session 50–60 min of cycling with intensities varying from under the aerobic threshold to over the anaerobic threshold	3–4 sets, 40–90% 1RM, 1–3 min rest between sets and exercises, 6–20 reps averaged length 60–90 min, including leg press, knee extension, bilateral or unilateral knee flexion, bench press, triceps pushdown, lateral pull-down, sit-up exercises for the trunk flexors or another exercise for the trunk extensors, and bilateral/unilateral elbow flexion exercise or leg adduction/abduction exercise	Supervised	2	21 weeks
Sillanpää et al., 2008 [95]	AT:1st cycle: 30 min under and above their aerobic threshold 2nd cycle: 45 min (15 min under the level of aerobic threshold, 10 min between the aerobic-anaerobic thresholds, 5 min above anaerobic threshold, and 15 min again under the aerobic threshold) 3rd cycle: 60 min (30 min under the level of aerobic threshold, 2 × 10 min between the aerobic-anaerobic thresholds, 2 × 5 min above anaerobic threshold); every other training session included 90 min of cycling at a steady pace under the aerobic threshold including bicycle ergometer RT:40–90% 1RM, 60–90 min including leg press, knee extension, bench press, triceps pushdown, or lateral pull-down, sit-up, bilateral/unilateral elbow flexion, or leg adduction/abduction	Separate	1st cycle: 30 min under and above their aerobic threshold 2nd cycle: 45 min (15 min under the level of aerobic threshold, 10 min between the aerobic and anaerobic thresholds, 5 min above the anaerobic threshold, and 15 min again under the aerobic threshold) 3rd cycle: 60 min (30 min under the level of aerobic threshold, 2 × 10 min between the aerobic and anaerobic thresholds, 2 × 5 min above the anaerobic threshold); every other training session include 90 min of cycling at a steady pace under the aerobic threshold including bicycle ergometer	40–90% 1RM, 60–90 min including leg press, knee extension, bench press, triceps pushdown, or lateral pull-down, sit-up, and bilateral/unilateral elbow flexion or leg adduction/abduction	Supervised	CT: 4 AT: 2 RT: 2	21 weeks
Sparks et al., 2013 [96]	AT: (10 kcal/kg body weight/week) at 50–80% VO_2peak_ RT: 1 set including bench press, seated row, shoulder press, lat pull-down, abdominal crunches, back extensions, leg press, extension, and flexion, 10–12 reps, 45–50 min	Separate	150 min/week moderate intensity exercise (12 kcal/kg body weight/week) at 50–80% VO_2peak_	2 sets (bench press, seated row, shoulder press, lat pull-down, abdominal crunches and back extensions) 3 sets (leg press, extension, and flexion), 10–12 reps, 45–50 min	Supervised	3	9 months
Swift et al., 2012 [97]	AT: (10 kcal/kg body weight/week), 50–80% VO_2max_ RT: 1 set, 10–12 reps, including bench press, seated row, shoulder press, lat pull-down, abdominal crunches, back extensions, leg press, extension, and flexion,	Separate	(12 kcal/kg body weight/week), 50–80% VO_2max_	2 sets, 10–12 reps (bench press, seated row, shoulder press, lat pull-down, abdominal crunches and back extensions), 3 sets (leg press, extension, and flexion),	Supervised	3	9 months
Theodorou et al., 2016 [98]	AT: 2 sets, (10 min each), 60–85% HR_max/peak_ running treadmill or cycling ergometer RT: 1 set, 12–15 reps, 60% 1RM, including abdominal, rotary torso, shoulder press, leg extension, leg curl, pulley, chest press, and leg press	Same	40 min, 60–85% HR_max/peak_. treadmill/cycling	2 sets, 12–15 reps, 60% 1RM, including abdominal, rotary torso, shoulder press, leg extension, leg curl, pulley, chest press, and leg press	Supervised	3	8 months
Timmons et al., 2018 [99]	AT: 3 × 4 min, 80% HR_max/peak_, 1 min recovery RT: 2 sets, 15 reps, 60% 1RM, including leg press, seated row, chest press, lat pull-down, leg extension, and tricep dips	Same	6 × 4 min, 80% HR_max/peak_, 1 min recovery, cross trainer or cycle ergometer	4 sets, 15 reps, 60% 1RM, including leg press, seated row, chest press, lat pull-down, leg extension, and tricep dips	Supervised	3	12 weeks
Zhou et al., 2022 [100]	AT: 50 min, 40–60% MHR treadmill/cycling/elliptical RT: 3 sets, 10–13 reps, 45–65% 1RM, 2 min rest between sets, 50 min, including chest press, back extension, shoulder press, leg press, leg extension/leg flexion, bicep curl/triceps extension, abdominal crunch, lat pull-down, and seated overhead press using dumbbells	Separate	50 min, 40–60% MHR treadmill/cycling/elliptical	3 sets, 10–13 reps, 45–65% 1RM, 2 min rest between sets, 50 min, including chest press, back extension, shoulder press, leg press, leg extension/leg flexion, bicep curl/triceps extension, abdominal crunch, lat pull-down, and seated overhead press using dumbbells	Supervised	3	12 weeks

Abbreviations: AT: aerobic training; CT: concurrent training; HR_AT_: heart rate corresponding to anaerobic threshold; HR_max/peak_: maximal or peak heart rate; HRR: heart rate reserve; MHR: maximal heart rate; ND: not described; RPE: Borg rating of perceived exertion; reps: repetitions; RT: resistance training; VO_2max/peak_: maximal or peak oxygen uptake; VT_1_: first ventilatory or aerobic threshold; VT_2_: second ventilatory or anaerobic threshold; 1RM: one repetition maximum.

**Table 3:** Summary of meta-analysis.

	Outcome	Trials	WMD or SMD (95% CI)	*p*-Value
CT vs. AT	Body weight	31	WMD: 0.31 kg (95% CI: 0.09 to 0.54)	0.006
	BMI	27	WMD: 0.03 kg·m^2^ (95% CI: −0.00 to 0.07)	0.11
	Body fat%	33	WMD: −0.30% (95% CI: −0.69 to 0.08)	0.13
	Fat mass	22	WMD: −0.01 kg (95% CI: −0.35 to 0.31)	0.91
	Waist circumference	20	WMD: −0.33 cm (95% CI: −0.90 to 0.23)	0.24
	Visceral fat	5	SMD: −0.02 (95% CI: −0.27 to 0.22)	0.84
	Lean body mass	27	WMD: 0.51 kg (95% CI: 0.29 to 0.73)	0.001
	Muscle mass/volume	10	SMD: 0.22 (95% CI: −0.01 to 0.45)	0.06
	CSA	7	SMD: 0.21 (95% CI: −0.02 to 0.44)	0.07
CT vs. RT	Body weight	23	WMD: −0.39 kg (95% CI: −0.96 to 0.16)	0.16
	BMI	21	WMD: −0.19 kg·m^2^ (95% CI: −0.43 to 0.04)	0.10
	Body fat%	21	WMD: −0.25% (95% CI: −0.74 to 0.23)	0.36
	Fat mass	13	WMD: −0.56 kg (95% CI: −1.23 to 0.10)	0.09
	Waist circumference	15	WMD: 0.09 cm (95% CI: −0.82 to 1.01)	0.83
	Lean body mass	12	WMD: −0.10 kg (95% CI: −0.61 to 0.41)	0.69
	Muscle mass/volume	7	SMD: −0.09 (95% CI: −0.38 to 0.19)	0.52
	CSA	5	SMD: −0.15 (95% CI: −0.40 to 0.10)	0.25

Abbreviations: AT: aerobic training; BMI: body mass index; CIs: 95% confidence intervals; CSA: muscle cross-sectional area; CT: concurrent training; RT: resistance training; SMD: standardized mean differences; WMD: weighted mean differences.

## Data Availability

The original contributions presented in this study are included in the article/Supplementary Material. Further inquiries can be directed to the corresponding authors.

## Supplementary Materials

The following supporting information can be downloaded at: https://www.mdpi.com/article/10.3390/healthcare13070776/s1: Table S1. Search strategy; Table S2. Risk of bias assessment. Table S3. Means and SD or Mean difference and SD (main or estimated) for body weight in CT vs. AT. Table S4. Means and SD or Mean difference and SD (main or estimated) for BMI in CT vs. AT. Table S5. Means and SD or Mean difference and SD (main or estimated) for fat percentage in CT vs. AT. Table S6. Means and SD or Mean difference and SD (main or estimated) for fat mass in CT vs. AT. Table S7. Means and SD or Mean difference and SD (main or estimated) for waist circumference in CT vs. AT. Table S8. Means and SD or Mean difference and SD (main or estimated) for visceral fat in CT vs. AT. Table S9. Means and SD or Mean difference and SD (main or estimated) for lean body mass in CT vs. AT. Table S10. Means and SD or Mean difference and SD (main or estimated) for muscle mass/volume in CT vs. AT. Table S11. Means and SD or Mean difference and SD (main or estimated) for CSA in CT vs. AT. Table S12. Means and SD or Mean difference and SD (main or estimated) for weight in CT vs. RT. Table S13. Means and SD or Mean difference and SD (main or estimated) for BMI in CT vs. RT. Table S14. Means and SD or Mean difference and SD (main or estimated) for fat percentage in CT vs. RT. Table S15. Means and SD or Mean difference and SD (main or estimated) for fat mass in CT vs. RT. Table S16. Means and SD or Mean difference and SD (main or estimated) for waist circumference in CT vs. RT. Table S17. Means and SD or Mean difference and SD (main or estimated) for lean body mass in CT vs. RT. Table S18. Means and SD or Mean difference and SD (main or estimated) for muscle mass/volume in CT vs. RT. Table S19. Means and SD or Mean difference and SD (main or estimated) for CSA in CT vs. RT. Table S20. Checklist item. Reference [121] is cited in the supplementary materials.
