# Supplementary material for: The Effects of Concurrent Training Versus Aerobic or Resistance Training Alone on Body Composition in Middle-Aged and Older Adults: A Systematic Review and Meta-Analysis
Source: Healthcare (Basel). 2025 Mar 31;13(7):776. doi: 10.3390/healthcare13070776 (PMC11989159; doi:10.3390/healthcare13070776)
Supplement: Supplementary file 1 [file healthcare-13-00776-s001.zip › healthcare-3483978-supplementary.pdf]

Supplementary Table S1. Search strategy

Databases	Search strategy
PubMed	(("concurrent"[All Fields] OR "concurrently"[All Fields] OR "concurrents"[All Fields] OR ("combinable"[All Fields] OR "combinated"[All Fields] OR "combination"[All Fields] OR "combinational"[All Fields] OR "combinations"[All Fields] OR "combinative"[All Fields] OR "combine"[All Fields] OR "combined"[All Fields] OR "combines"[All Fields] OR "combining"[All Fields]) OR ("combinable"[All Fields] OR "combinated"[All Fields] OR "combination"[All Fields] OR "combinational"[All Fields] OR "combinations"[All Fields] OR "combinative"[All Fields] OR "combine"[All Fields] OR "combined"[All Fields] OR "combines"[All Fields] OR "combining"[All Fields])) AND ("strength training"[All Fields] OR "strength exercise"[All Fields] OR "resistance training"[All Fields] OR "resistance exercise"[All Fields] OR "endurance training"[All Fields] OR "endurance exercise"[All Fields] OR "aerobic training"[All Fields] OR "aerobic exercise"[All Fields]) AND ("old"[All Fields] OR ("aged"[MeSH Terms] OR "aged"[All Fields] OR "elderly"[All Fields] OR "elderlies"[All Fields] OR "elderly s"[All Fields] OR "elderlys"[All Fields]) OR ("older"[All Fields] OR "olders"[All Fields]) OR ("aged"[MeSH Terms] OR "aged"[All Fields])) AND ("randomized control trial"[All Fields] OR "randomized clinical trial"[All Fields] OR "randomized"[All Fields] OR "random*"[All Fields])) AND ((humans[Filter]) AND (english[Filter]))
Scopus	(TITLE-ABS-KEY (concurrent OR combined OR combination) AND TITLE-ABS-KEY ("strength training" OR "strength exercise" OR "resistance training" OR "resistance exercise" OR "endurance training" OR "endurance exercise" OR "aerobic training" OR "aerobic exercise") AND TITLE-ABS-KEY (old OR elderly OR older OR aged) AND TITLE-ABS-KEY ("randomized control trial" OR "randomized clinical trial" OR "randomized" OR "random*")) AND (LIMIT-TO (DOCTYPE , "ar")) AND (LIMIT-TO (LANGUAGE , "English")) AND (LIMIT-TO (EXACTKEYWORD , "Human"))
Web of science	(((TS=(concurrent OR combined OR combination)) AND TS=("strength training" OR "strength exercise" OR "resistance training" OR "resistance exercise" OR "endurance training" OR "endurance exercise" OR "aerobic training" OR "aerobic exercise")) AND TS=(old OR elderly OR older OR aged)) AND TS=("randomized control trial" OR "randomized clinical trial" OR "randomized" OR "random*") and Article (Document Types) and English (Languages)

Supplementary Table S2. Risk of bias assessment

First author, year	Criteria 1	Criteria 2	Criteria 3	Criteria 4	Criteria 5	Criteria 6	Criteria 7	Criteria 8	Criteria 9	Score
Ahtiainen et al, 2009 [30]	✓	✓	x	✓	x	✓	x	✓	✓	6
Aminilari et al, 2017 [31]	✓	✓	x	✓	x	✓	x	✓	✓	6
Balducci et al, 2010 [52]	✓	✓	x	✓	✓	✓	✓	✓	✓	8
Bernard et al, 1999 [53]	x	✓	x	✓	✓	x	x	✓	✓	5
Bouchla et al, 2011 [54]	✓	✓	x	✓	x	✓	x	✓	✓	6
Boulé et al, 2013 [55]	✓	✓	x	✓	x	✓	x	✓	✓	6
Burich et al, 2015 [56]	✓	✓	✓	x	✓	✓	✓	✓	✓	8
Cadore et al, 2010 [32]	✓	✓	x	✓	✓	x	x	✓	✓	6
Campos et al, 2013 [33]	x	✓	x	✓	x	x	x	✓	✓	4
Chang et al, 2023 [57]	✓	✓	✓	✓	x	✓	x	✓	✓	7
Chen et al, 2017 [34]	✓	✓	x	✓	✓	x	x	✓	✓	6
Choi et al, 2021 [58]	x	✓	x	✓	x	✓	x	✓	✓	5
Church et al, 2010 [35]	✓	✓	✓	✓	✓	✓	✓	✓	✓	9
Cortz-Cooper et al, 2007 [59]	✓	✓	x	✓	x	✓	x	✓	✓	6
Cuff et al, 2003 [60]	✓	✓	x	✓	x	✓	x	✓	✓	6
Davidson et al, 2009 [36]	✓	✓	x	✓	✓	✓	✓	✓	✓	8
Delaney et al, 2014 [61]	✓	✓	✓	✓	✓	✓	✓	✓	✓	9
Delecluse et al, 2004 [62]	✓	✓	x	✓	x	✓	x	✓	✓	6
Do Amaral et al, 2021 [63]	✓	✓	x	✓	x	x	x	✓	✓	5
Feiereisen et al, 2007 [64]	✓	✓	x	✓	x	✓	x	✓	✓	6
Gayda et al, 2009 [65]	✓	✓	x	?	x	✓	x	x	✓	4
Gonzalo-Encabo et al, 2020 [66]	✓	✓	✓	✓	x	✓	x	✓	✓	7
Hansen et al, 2011 [67]	✓	✓	x	x	✓	x	x	✓	✓	6
Ho et al, 2012 [37]	✓	✓	x	✓	x	x	x	✓	✓	5
Irving et al, 2015 [38]	✓	✓	x	✓	x	✓	x	✓	✓	7
Izquterdo et al, 2004 [39]	✓	✓	x	✓	x	✓	x	✓	✓	6
Jorge et al, 2011 [68]	✓	✓	x	✓	x	✓	x	✓	✓	6
Kadoglou et al, 2013 [40]	✓	✓	x	✓	✓	✓	x	✓	✓	8

Karavirta et al, 2011 [41]	x	✓	x	✓	x	✓	x	✓	✓	6
Kim et al, 2018 [69]	✓	✓	✓	✓	x	x	x	✓	✓	6
Kobayashi et al, 2023 [42]	✓	✓	✓	✓	✓	x	✓	✓	✓	8
Lee et al, 2015 [70]	✓	✓	x	✓	x	x	x	✓	✓	5
Lima et al, 2017 [71]	✓	✓	✓	✓	x	✓	x	✓	✓	7
Marzolini et al, 2018 [72]	✓	✓	✓	✓	✓	✓	x	✓	✓	8
Marzolini et al, 2008 [73]	✓	✓	✓	x	✓	x	x	✓	✓	6
et Moreno-Cabañas al, 2021 [74]	✓	✓	x	✓	x	✓	x	✓	✓	6
Pedralli et al, 2020 [75]	✓	✓	✓	✓	✓	✓	x	✓	✓	8
Piralaiy et al, 2021 [43]	✓	✓	x	✓	x	x	x	✓	✓	5
Rossi et al, 2016 [76]	✓	✓	x	✓	x	x	x	✓	✓	5
Rossi et al, 2018 [77]	✓	✓	x	✓	x	✓	x	✓	✓	6
Ruangthai et al, 2019 [44]	✓	✓	x	✓	x	x	x	✓	✓	5
Schroeder et al, 2019 [45]	✓	✓	x	✓	✓	✓	✓	✓	✓	8
Scott et al, 2021[46]	✓	✓	✓	✓	✓	✓	✓	✓	✓	9
Sénéchal et al, 2013 [78]	✓	✓	x	✓	x	x	x	✓	✓	5
Seo et al, 2010 [79]	✓	✓	x	✓	x	✓	x	✓	✓	6
Sigal et al, 2007 [80]	✓	✓	✓	✓	x	✓	x	✓	✓	7
Sillanpää et al, 2010 [81]	✓	✓	x	✓	x	✓	x	✓	✓	6
Sillanpää et al, 2008 [82]	✓	✓	x	✓	x	✓	x	✓	✓	6
Sparks et al, 2013 [83]	x	✓	x	✓	x	✓	x	✓	✓	5
Swift et al, 2012 [84]	✓	✓	x	✓	✓	x	x	✓	✓	6
Theodorou et al, 2016 [85]	✓	✓	x	✓	x	✓	x	✓	✓	6
Timmons et al, 2018 [86]	✓	✓	✓	x	✓	✓	x	✓	✓	7
Zhou et al, 2022 [87]	✓	✓	✓	✓	x	✓	x	✓	✓	7

(1) Eligibility Criteria specified, (2) Random allocation of participants, (3) Allocation concealed, (4) Groups similar at baseline, (5) Assessors blinded, (6) Outcome measures assessed in 85% of participants, (7) Intention to treat analysis, (8) Reporting of between group statistical comparison, (9) Point measures and measures of variability reported for main effects. 'low (✓), 'high (x) and unclear (?)

Supplementary Table S3. Means and SD or Mean difference and SD (main or estimated) for body weight in CT vs. AT

Study name	CT				AT			
	Pre		Post		Pre		Post	
	Mean	SD	Mean	SD	Mean	SD	Mean	SD
	Mean change		SD change		Mean change		SD change	
Ahtiainen et al, 2009	80	7	78	6	75	7	74	7
Aminilari et al, 2017	69.79	6.11	68.3	6.75	72.64	12.89	71.94	13.19
Balducci et al, 2010	84.6	2.9	85.2	2.9	79.5	3.6	79	3.5
Boulé et al, 2013	0.2		0.47		-0.3		0.5	
Burich et al, 2015	80.3	18.3	78.5	18	69.5	12.9	69.1	12.5
Cadore et al, 2010	85.3	11.9	89.7	10.7	79.1	13.9	78	12.4
Campos et al, 2013 a (AS)	81.1	16.1	80	15.1	67.4	18	67.1	17.8
Campos et al, 2013 b (SA)	66.7	14.2	65.8	13.3	67.4	18	67.1	17.8
Chang et al, 2023	-0.5		0.94		0.05		1.12	
Chen et al, 2017	64.5	10.1	63.9	9.9	62.8	9.4	62.2	9.4
Choi et al, 2021	70.86	4.12	67.23	2.07	69.59	2.93	66.59	2.06
Cuff et al, 2003	-2.9		4.11		-1.2		2.1	
Church et al, 2010	-1.5		3.49		-0.8		3.19	
Davidson et al, 2009	-2.31		1.89		-2.77		1.8	
Gayda et al, 2009	86	8	84	7	85	15	84	14
Gonzalo-Encabo et al, 2020	83.7	11.29	84.21	11.49	84.6	8.6	82.88	8.42
Ho et al, 2012	90	16.49	88.4	14.84	91.9	15.87	91	15.49
Izquterdo et al, 2004	74.7	7.5	74.8	7.5	76.7	7.4	76.9	6.8
Lee et al, 2015	57.71	4.06	57.47	3.9	56.16	3.96	54.72	4.48
Moreno-Cabañas et al, 2021	93	18.3	92	18.4	93.7	11.2	93.1	11.6
Pedralli et al, 2020	78.2	23.3	77	23.9	79	12.9	77.8	12.9
Piralaiy et al, 2021	88.76	14.31	87.03	13.91	91.74	13.89	89.69	12.8
Rossi et al, 2016	64.6	10.6	64.3	10.7	66.8	10.9	66.3	11
Ruangthai et al, 2019	60.2	9.1	59.8	9.4	58.5	7.7	58.2	7.6
Schroeder et al, 2019								
Seo et al, 2010	59.8	7.7	59.2	7.1	68.3	12.9	66.9	12.8
Sigal et al, 2007	101.9	30.4	99.3	30.4	103.5	31	100.9	30.2
Sillanpaa et al, 2008								
Swift et al, 2012								
Theodorou et al, 2016	85.2	8.13	81.5	8.13	87.5	11.23	84.8	8.52
Timmons et al, 2018	80	7	78	6	75	7	74	7

Supplementary Table S4. Means and SD or Mean difference and SD (main or estimated) for BMI in CT vs. AT

Study name	CT				AT			
	Pre		Post		Pre		Post	
	Mean	SD	Mean	SD	Mean	SD	Mean	SD
	Mean change		SD change		Mean change		SD change	
Aminilari et al, 2017	29.01	2.57	28.37	2.57	30.03	5.48	29.73	5.57
Balducci et al, 2010	30.5	0.9	30.2	0.8	29.4	1.1	29.1	1.1
Chang et al, 2023	-0.19		0.39		0.06		0.57	
Chen et al. 2017	27.2	2.9	26.9	2.8	26.8	3.8	26.6	3.9
Choi et al, 2021	24.92	2.55	23.8	2.43	25.1	2.78	23.51	1.95
Davidson et al. 2009	-0.84		0.68		-0.96		0.65	
Ho et al, 2012	33.3	4.94	32.8	4.53	32.7	5.03	32.4	4.64
Izquterdo et al, 2004	25.2	8.7	25.1	9.2	27.6	2.7	27.6	2.4
Jorge et al, 2011	31.23	3.88	31.1	3.53	29.3	2.2	29.08	2.42
Kadoglou et al, 2013	-0.22		0.08		-0.24		0.05	
Lee et al, 2015	24.37	1.24	24.27	1.17	24	0.93	23.38	1.08
Lima et al, 2017	27.6	3.4	27	1.36	28.9	3.5	28.05	3.42
Marzolini et al, 2018	27.78	4.3	28.1	4.5	25.32	5.1	25.6	5.3
Moreno-Cabañas et al, 2021	32.7	5.2	32.3	5.2	32	4.1	31.8	4
Pedralli et al, 2020	27.9	5.5	27.3	5.8	29.8	4.1	29.3	4.2
Piralaiy et al, 2021	30.36	3.02	29.81	2.56	30.39	3.36	29.45	3.21
Rossi et al, 2016	28.3	2.7	28.3	2.8	28.4	2.9	28.1	2.9
Ruangthai et al, 2019	24.1	2	24	2.1	23.8	2.4	23.7	2.4
Schroeder et al, 2019	0.2		0.5		-0.3		0.68	
Seo et al, 2010	24	1.9	23.8	1.7	27.4	3.4	26.8	3.3
Sigal et al, 2007	35	9.6	34.2	9.6	35.6	10.1	34.8	10.1
Sillanpaa et al, 2010	25	3.1	24.8	2.9	25.1	2.6	24.8	2.5
Sillanpaa et al, 2008	-0.1		0.18		-0.5		0.51	
Sparks et al, 2013	-0.74		1.03		-0.49		1.03	
Theodorou et al, 2016	29.8	3.48	28.6	3.48	31.1	2.71	30.1	2.71
Zhou et al, 2022 a	24.29	3.98	24.21	3.9	23.82	2.96	23.9	2.73
Zhou et al, 2022 b	24.6	2.89	24.45	2.89	23.82	2.96	23.9	2.73

Supplementary Table S5. Means and SD or Mean difference and SD (main or estimated) for fat percentage in CT vs. AT

Study name	CT				AT			
	Pre		Post		Pre		Post	
	Mean	SD	Mean	SD	Mean	SD	Mean	SD
	Mean change		SD change		Mean change		SD change	
Ahtiainen et al, 2009	25	3	23	3	26	3	24	2
Aminilari et al, 2017	36.29	3.98	34.31	4.07	37.03	4.22	35.39	4.32
Balducci et al, 2010	32.2	1.8	31.6	1.7	32.2	2.4	32.4	2.4
Burich et al, 2015	38.4	6	33.8	7.4	33.2	7.7	28.9	6.1
Cadore et al, 2010	26.2	3.9	28.8	2.9	28.9	4.5	27.1	5
Campos et al, 2013 a (AS)	33.2	8	31	7.1	35.6	11.3	31.2	7
Campos et al, 2013 b (SA)	39.6	6.8	33.6	5.4	35.6	11.3	31.2	7
Chang et al, 2023	2.11		4.31		1.46		5.23	
Chen et al. 2017	39.7	5.8	38.3	5.5	40	4.4	38.6	4.9
Choi et al, 2021	24.83	2.7	23.64	2.83	24.41	2.92	22.86	2.05
Delecluse et al, 2004 a	27.4	5.9	27.3	4.8	28.2	5.8	27.6	5.6
Delecluse et al, 2004 b	28	5.8	27.9	5.9	28.2	5.8	27.6	5.6
Gayda et al, 2009	27	2	28	3	28	3	30	4
Ho et al, 2012	45.8	6.59	44.8	7.42	44.6	7.35	44.1	6.97
Irving et al, 2015	-1.3		2.4		-1.3		6.3	
Izquterdo et al, 2004	21.2	2.3	20.8	2.5	21.8	4.6	21.8	4.7
Kadoglou et al, 2013	-1.97		0.55		-1.18		0.49	
Lee et al, 2015	27.79	1.84	26.19	2.55	28.24	1.23	27.15	1.93
Marzolini et al, 2018	30.4	6.4	29.7	6.6	26.9	6.8	27.1	6.6
Marzolini et al, 2008 a	-2.74		3.05		-0.08		0.93	
Marzolini et al, 2008 b	-2.01		3.13		-0.08		0.93	
Piralaiy et al, 2021	30.02	7.94	25.97	6.49	29.55	7.12	24.18	7.45
Rossi et al, 2016	42.5	5	40.9	5.4	43.9	5.5	42.7	5.4
Ruangthai et al, 2019	32.3	6.3	31.4	5.9	32.3	7.6	31.7	6.7
Schroeder et al, 2019	-0.5		0.9		-0.5		1.06	
Seo et al, 2010	31.7	3	30.4	3.1	33.6	3.8	32.8	3.5
Sigal et al, 2007	36	9.6	35	9.6	37	9.3	36.3	9.3
Sillanpaa et al, 2010	34.9	6.5	32.9	6.6	37.7	5.1	36.3	5.3
Sillanpaa et al, 2008	0.2		0.27		-0.3		0.6	
Sparks et al, 2013	-1.4		1.73		-0.1		1.73	
Swift et al, 2012	-1.05		1.763		-0.42		1.942	
Theodorou et al, 2016	32.9	2.32	31.9	1.93	33.6	2.71	32.2	2.71
Timmons et al, 2018	-2		3.51		-3.5		3.4	

Supplementary Table S6. Means and SD or Mean difference and SD (main or estimated) for fat mass in CT vs. AT

Study name	CT				AT			
	Pre		Post		Pre		Post	
	Mean	SD	Mean	SD	Mean	SD	Mean	SD
	Mean change		SD change		Mean change		SD change	
Bouchla et al, 2011	26.5	9.3	27.4	9.3	26.8	7	26.4	5.6
Cadore et al, 2010	22.7	6.3	25.1	5.7	23	5.6	21.4	5.9
Campos et al, 2013 a	26.1	4.2	24.2	4.7	22.6	2.9	20.2	3.2
Campos et al, 2013 b	26.8	9.4	22.6	8.2	22.6	2.9	20.2	3.2
Chen et al, 2017	25.7	6	24.6	5.8	25.3	5.6	24.2	5.8
Church et al, 2010	-1.7		2.62		-0.6		2.34	
Davidson et al, 2009	-3.38		2.01		-3.03		2.37	
Gonzalo-Encabo et al, 2020	38.48	8.23	36.65	8.06	37.44	7.15	35.31	7.38
Hansen et al, 2011	16.8	5.3	16	5.3	19.4	6.3	18.4	6
Ho et al, 2012	39.3	7.42	37.7	7.42	39.3	9.68	38.6	9.68
Irving et al, 2015	-2.2		4.5		-1.9		2.32	
Kobayashi et al, 2023	-0.62		1.8		-0.7		1.26	
Lee et al, 2015	16.3	1.62	15.09	2.01	15.9	1.78	14.91	2.11
Lima et al, 2017	24.5	5.9	21.8	3.9	26.7	5.8	25.4	5.6
Moreno-Cabañas et al, 2021	32.5	9.6	31.5	10.1	31.8	8.2	31.2	8.4
Rossi et al, 2016	27.9	7.2	26.6	7.2	31.8	11.3	28.6	7.1
Rossi et al, 2018	24	4.5	23.1	5.3	30.1	5.1	28.8	4.5
Ruangthai et al, 2019	19.4	4.3	18.7	4.2	18.8	4.9	18.4	4.4
Schroeder et al, 2019	-0.1		0.2		-0.9		1.26	
Sigal et al, 2007	37.6	19.2	35.7	19.2	39.2	19.4	37.6	19.4
Swift et al, 2012	-1.6		2.62		-0.94		2.817	
Timmons et al, 2018	-2.6		3.84		-2.2		3.84	

Supplementary Table S7. Means and SD or Mean difference and SD (main or estimated) for waist circumference in CT vs. AT

Study name	CT				AT			
	Pre		Post		Pre		Post	
	Mean	SD	Mean	SD	Mean	SD	Mean	SD
	Mean change		SD change		Mean change		SD change	
Balducci et al, 2010	102	2.8	97	2.4	99.8	2.7	97.6	2.6
Boulé et al, 2013		-2.1		4.02		-1.6		3.31
Campos et al, 2013 a (AS)	103	10.6	103	10.4	91.4	10.5	90.3	12.4
Campos et al, 2013 b (SA)	93.5	12	96	11.8	91.4	10.5	90.3	12.4
Chang et al, 2023		-2.18		5.82		-2.68		3.33
church et al, 2010		-2.8		3.93		-1.5		4.04
Davidson et al, 2009		-4.61		2.69		-5.08		2.51
Delecluse et al, 2004 a	98.9	9.5	96.5	9.3	97.5	9.6	94.9	9.3
Delecluse et al, 2004 b	97	10.9	94.9	9.8	97.5	9.6	94.9	9.3
Ho et al, 2012	102	13.19	99.6	12.36	103.7	10.06	101.6	11.23
Lima et al, 2017	88.9	7.7	86.7	7.6	90.1	12	88.5	11.9
Moreno-Cabañas et al, 2021	109.2	13	106.4	13.4	109.4	8.4	109.5	8
Pedralli et al, 2020	89.6	14.5	86.5	15	92.4	8.1	88.3	9.2
Schroeder et al, 2019		0.9		1.8		0.4		0.77
Seo et al, 2010	81.9	4.7	77.6	5.4	87.9	8.6	84.7	7.9
Sigal et al, 2007	112	24	108	24	113	23	110	23
Sillanpaa et al, 2008		-1.8		1.98		-2.7		1.99
Swift et al, 2012		-2.49		4.33		-1.68		4.487
Zhou et al, 2022 a	90.93	12.5	87.5	12.5	91.56	7.81	89.68	7.81
Zhou et al, 2022 b	91.56	8.12	87.81	7.81	91.56	7.81	89.68	7.81

Supplementary Table S8. Means and SD or Mean difference and SD (main or estimated) for visceral fat in CT vs. AT

Study name	CT				AT			
	Pre		Post		Pre		Post	
	Mean	SD	Mean	SD	Mean	SD	Mean	SD
	Mean change		SD change		Mean change		SD change	
Chang et al, 2023		5.5		11.26		7.21		26.03
Chen et al, 2017	111.9	31.5	107.4	30.6	122.2	35.7	118.4	30.9
Cuff et al, 2003		-26.3		23.4		-8.8		16.2
Davidson et al, 2009		-0.35		0.28		-0.43		0.43
Sigal et al, 2007	246	159	224	159	257	161	244	161

Supplementary Table S9. Means and SD or Mean difference and SD (main or estimated) for lean body mass in CT vs. AT

Study name	CT				AT			
	Pre		Post		Pre		Post	
	Mean	SD	Mean	SD	Mean	SD	Mean	SD
	Mean change		SD change		Mean change		SD change	
Ahtiainen et al, 2009	60	3	61	4	56	5	56	5
Bouchla et al, 2011	53.2	9.8	53.7	10.7	55.1	6.3	55.5	5.1
Choi et al, 2021	44.72	5.45	46.4	4.58	43.08	3.09	42.37	2.13
church et al, 2010	0		0.21		-0.5		1.91	
Delecluse et al, 2004 a	61.1	6.8	60.9	6.8	57.8	4.7	57.7	5.1
Delecluse et al, 2004 b	56.6	5.6	56.6	5.3	57.8	4.7	57.7	5.1
Gayda et al, 2009	62	5	61	6	61	8	59	10
Gonzalo-Encabo et al, 2020	47.9	3.54	48.48	3.96	47.6	1.95	47.7	1.66
Hansen et al, 2011	43.6	6.5	44.4	6.5	42.7	5.6	42.7	5.6
Irving et al, 2015	1.5		2.1		0.3		1.65	
Izquterdo et al, 2004	58.6	5.3	58.8	5.3	59.7	3.4	60.8	4.6
Kobayashi et al, 2023	-0.23		1.22		-0.37		1.26	
Lee et al, 2015	41.41	3.13	42.38	2.78	40.26	2.25	39.81	2.5
Lima et al, 2017	44.3	8.3	44.1	6.9	42.2	7.8	41.9	6.6
Marzolini et al, 2008 a	0.84		0.87		0.38		0.59	
Marzolini et al, 2008 b	1.51		1.01		0.38		0.59	
Marzolini et al, 2018	51.2	12.6	52.5	12.4	47.2	9	47.5	9.2
Moreno-Cabañas et al, 2021	60.5	12.3	60.4	12.5	62	8.7	61.9	8.9
Rossi et al, 2016	34.4	4.4	35.3	4.7	34.7	5	35.3	5.1
Rossi et al, 2018	33.7	4	34.8	4	34	3.3	34.4	3.3
Schroeder et al, 2019	0.8		1.4		-0.3		0.48	
Sigal et al, 2007	63.9	13.6	63.2	13.6	64	13.9	63	13.9
Sillanpaa et al, 2008	1.63		2.41		0.57		1.87	
Sillanpaa et al, 2010	41.433	3.495	42.299	3.204	39.809	3.866	40.109	3.492
Sparks et al, 2013	-0.3		5.19		-0.5		1.73	
Swift et al, 2012	0.04		2.057		-0.44		2.195	
Timmons et al, 2018	0.4		0.87		0.5		0.98	

Supplementary Table S10. Means and SD or Mean difference and SD (main or estimated) for muscle mass/volume in CT vs. AT

Study name	CT				AT			
	Pre		Post		Pre		Post	
	Mean	SD	Mean	SD	Mean	SD	Mean	SD
	Mean change		SD change		Mean change		SD change	
Aminilari et al, 2017	40.18	3.86	41.72	4.06	41.74	5.16	43.24	5.25
Campos et al, 2013 a (AS)	28.2	13.4	29.3	12.5	22	16.2	23.9	13
Campos et al, 2013 b (SA)	15.7	3.9	19.2	2.2	22	16.2	23.9	13
Chen et al. 2017	20.7	4	20.9	3.7	20	3.3	20.2	2.3
Davidson et al, 2009	0.62		0.86		-0.06		1.04	
Delaney et al, 2014	25.43	5.42	25.64	5.52	23.72	6.77	23.47	6.62
Hansen et al, 2011	43.6	6.5	44.4	6.5	42.7	5.6	42.7	5.6
Ruangthai et al, 2019	21.9	5.1	22.1	5	21.3	4.8	21.4	4.4
Feiereisen et al, 2007	7121	1437	7449	1388	6554	1298	6739	1107
Gayda et al, 2009	8.8	0.5	9.4	1.3	8.1	1.5	8.1	1.5

Supplementary Table S11. Means and SD or Mean difference and SD (main or estimated) for CSA in CT vs. AT

Study name	CT				AT			
	Pre		Post		Pre		Post	
	Mean	SD	Mean	SD	Mean	SD	Mean	SD
	Mean change		SD change		Mean change		SD change	
Ahtiainen et al, 2009	5021	829	5654	2214	5427	1135	5545	965
Bernard et al, 1999	7.56		6.41		2.69		5.58	
Cuff et al., 2003	5.9		6.32		0.9		6.3	
Izquterdo et al, 2004	39.61	4.8	43.49	5.42	46.21	9.19	48.07	9.61
Karavirta et al, 2011	8.46		34.88		7.98		30.22	
Irving et al, 2015	5.1		8.4		4		4.97	
Sigal et al, 2007	309	71	317	71	309	67	314	67

Supplementary Table S12. Means and SD or Mean difference and SD (main or estimated) for weight in CT vs. RT

Study name	CT				AT			
	Pre		Post		Pre		Post	
	Mean	SD	Mean	SD	Mean	SD	Mean	SD
	Mean change		SD change		Mean change		SD change	
Ahtiainen et al, 2009	80	7	78	6	80	5	79	6
Aminilari et al, 2017	69.79	6.11	68.3	6.75	72.44	6.37	70.92	7.03
Boulé et al, 2013	0.2		0.47		-0.1		0.21	
Cadore et al, 2010	85.3	11.9	89.7	10.7	80.8	12.2	80.8	12
Campos et al, 2013 a (AS)	81.1	16.1	80	15.1	64.8	11.9	64.5	11.8
Campos et al, 2013 b (SA)	66.7	14.2	65.8	13.3	64.8	11.9	64.5	11.8
Chen et al, 2017	64.5	10.1	63.9	9.9	70.3	11.2	69.7	10.6
Church et al, 2010	-1.5		3.49		-0.3		1.28	
Cortz-Cooper et al, 2007	76.3	16.28	76.6	16.62	76.4	10.81	77.3	10.09
Davidson et al, 2009	-2.31		1.89		-0.64		2.02	
Do Amaral et al, 2021	77.3	20.3	79.1	20.4	74.2	15.7	74.2	15.8
Ho et al, 2012	90	16.49	88.4	14.84	89.3	18	89.2	17.6
Izquterdo et al, 2004	74.7	7.5	74.8	7.5	81.3	11.3	80.1	9.7
Kim et al, 2018	57.07	8.03	56.42	8.36	60.05	8.1	59.68	8.12
Pedralli et al, 2020	78.2	23.3	77	23.9	81.4	22.3	81	22
Piralaiy et al, 2021	88.76	14.31	87.03	13.91	85.64	8.6	84.6	8.09
Ruangthai et al, 2019	60.2	9.1	59.8	9.4	52.3	7.6	51.9	3.3
Schroeder et al, 2019	0.9		1.8		-0.2		0.38	
Sigal et al, 2007	101.9	30.4	99.3	30.4	99.1	30.4	98	30.4
Sillanpaa et al, 2008	-0.6		1.976		-0.9		2.023	
Swift et al, 2012	-1.35		3.331		-0.03		3.06	
Theodorou et al, 2016	85.2	8.13	81.5	8.13	88.7	11.93	86.5	11.93
Timmons et al, 2018	-0.8		1.64		-0.1		0.1	

Supplementary Table S13. Means and SD or Mean difference and SD (main or estimated) for BMI in CT vs. RT

Study name	CT				AT			
	Pre		Post		Pre		Post	
	Mean	SD	Mean	SD	Mean	SD	Mean	SD
	Mean change		SD change		Mean change		SD change	
Aminilari et al, 2017	29.01	2.57	28.37	2.57	29.11	1.92	28.54	1.98
Chen et al. 2017	27.2	2.9	26.9	2.8	28.3	4.4	28.1	4.3
Cortz-Cooper et al. 2007	27	3.81	27.1	3.46	26.8	3.96	27.1	3.96
Davidson et al, 2009	-0.84		0.68		-0.26		0.65	
Do Amaral et al, 2021	31	7.9	31.5	7.9	30	6.1	30.1	5.1
Ho et al, 2012	33.3	4.94	32.8	4.53	33	5.2	33	5.2
Izquterdo et al, 2004	25.2	8.7	25.1	9.2	29.6	4.1	29.3	3.5
Jorge et al, 2011	31.23	3.88	31.1	3.53	32.07	3.79	30.76	5.03
Kadoglou et al, 2013			-0.22		0.08		0.13	
Kim et al, 2018	24.94	1.63	24.64	1.73	25.75	2.6	25.45	2.52
Pedralli et al, 2020	27.9	5.5	27.3	5.8	28.5	6.01	28.4	5.8
Piralaiy et al, 2021	30.36	3.02	29.81	2.56	28.79	2.59	28.42	2.5
Ruangthai et al, 2019	24.1	2	24	2.1	22.6	2.6	22.4	2.5
Schroeder et al, 2019	0.2		0.5		-0.1		0.29	
Sigal et al, 2007	35	9.6	34.2	9.6	34.1	9.6	33.7	9.6
Sillanpaa et al, 2008								
Sillanpaa et al, 2010	25	3.1	24.8	2.9	24.7	3.2	24.6	3
Sparks et al, 2013	-0.1		0.592		-0.3		0.643	
Theodorou et al, 2016	29.8	3.48	28.6	3.48	31.6	4.64	30.9	5.3
Zhou et al, 2022 a	24.29	3.98	24.21	3.9	25.85	3.05	25.85	2.89
Zhou et al, 2022 b	24.6	2.89	24.45	2.89	25.85	3.05	25.85	2.89

Supplementary Table S14. Means and SD or Mean difference and SD (main or estimated) for fat percentage in CT vs. RT

Study name	CT				AT			
	Pre		Post		Pre		Post	
	Mean	SD	Mean	SD	Mean	SD	Mean	SD
	Mean change		SD change		Mean change		SD change	
Ahtiainen et al, 2009	25	3	23	3	24	3	22	3
Cadore et al, 2010	26.2	3.9	28.8	2.9	27.3	2.7	27.9	2.8
Campos et al, 2013 a (AS)	33.2	8	31	7.1	40.1	12.8	38.3	15.5
Campos et al, 2013 b (SA)	39.6	6.8	33.6	5.4	40.1	12.8	38.3	15.5
Chen et al. 2017	39.7	5.8	38.3	5.5	39.7	5.6	38.8	6.3
Cortz-Cooper et al. 2007	41	10.39	40	10.39	39	10.81	38	10.81
Ho et al, 2012	45.8	6.59	44.8	7.42	43.7	5.2	43.2	5.6
Irving et al, 2015	-1.3		2.4		-0.9		2.84	
Izquterdo et al, 2004	21.2	2.3	20.8	2.5	24	5.7	22.2	4.3
Kadoglou et al, 2013	-1.97		0.55		-0.66		0.21	
Kim et al, 2018	36.06	2.64	37.18	2.74	35.78	3.8	36.45	3.3
Piralaiy et al, 2021	30.02	7.94	25.97	6.49	28	5.49	22.43	8.3
Ruangthai et al, 2019	32.3	6.3	31.4	5.9	33.4	5	32.3	5.3
Schroeder et al, 2019	-0.5		0.9		-0.2		0.38	
Sigal et al, 2007	36	9.6	35	9.6	35.9	9.6	35	9.6
Sillanpaa et al, 2010	34.9	6.5	32.9	6.6	35.4	7.5	34.3	7.7
Sillanpaa et al, 2008	0.2		0.27		0.3		0.49	
Sparks et al, 2013	-1.4		1.73		-1		1.69	
Swift et al, 2012	-1.05		1.763		-1.09		1.623	
Theodorou et al, 2016	32.9	2.32	31.9	1.93	33.5	1.65	32.4	0.66
Timmons et al, 2018	-2		3.51		-1.8		3.4	

Supplementary Table S15. Means and SD or Mean difference and SD (main or estimated) for fat mass in CT vs. RT

Study name	CT				AT			
	Pre		Post		Pre		Post	
	Mean	SD	Mean	SD	Mean	SD	Mean	SD
	Mean change		SD change		Mean change		SD change	
Cadore et al, 2010	22.7	6.3	25.1	5.7	22.2	5	25.6	6
Campos et al, 2013 a (AS)	26.1	4.2	24.2	4.7	27	13	26	15
Campos et al, 2013 b (SA)	26.8	9.4	22.6	8.2	27	13	26	15
Chen et al, 2017	25.7	6	24.6	5.8	27.9	6.8	27.3	7.5
church et al, 2010	-1.7		2.62		-1.4		2.78	
Davidson et al, 2009	-3.38		2.01		-1.56		2.16	
Ho et al, 2012	39.3	7.42	37.7	7.42	37.6	9.2	37.2	9.6
Irving et al, 2015	-2.2		4.5		-0.5		4.11	
Kobayashi et al, 2023	-0.62		1.8		-0.99		1.78	
Ruangthai et al, 2019	19.4	4.3	18.7	4.2	17.7	4.9	17.1	5
Schroeder et al, 2019	-0.1		0.2		-0.3		0.68	
Sigal et al, 2007	37.6	19.2	35.7	19.2	36.5	19.2	35.2	19.2
Timmons et al, 2018	-2.6		3.84		-0.4		0.76	

Supplementary Table S16. Means and SD or Mean difference and SD (main or estimated) for waist circumference in CT vs. RT

Study name	CT				AT			
	Pre		Post		Pre		Post	
	Mean	SD	Mean	SD	Mean	SD	Mean	SD
	Mean change		SD change		Mean change		SD change	
Boulé et al, 2013	-2.1		4.45		-3.9		3.95	
Campos et al, 2013 a (AS)	103	10.6	103	10.4	91.4	6.7	91.5	8.8
Campos et al, 2013 b (SA)	93.5	12	96	11.8	91.4	6.7	91.5	8.8
Church et al, 2010	-2.8		3.93		-1.9		3.85	
Davidson et al, 2009	-4.61		2.69		-3.18		2.68	
Do Amaral et al, 2021	104	16	101	15	104	12	101	12
Ho et al, 2012	102.2	13.19	99.6	12.36	104	12.8	101.4	13.2
Kim et al, 2018	84.76	3.5	81.11	5.2	86.91	7.97	82.54	7.89
Pedralli et al, 2020	89.6	14.5	86.5	15	92.7	16.5	90.7	15.1
Schroeder et al, 2019	0.9		1.8		-1.7		3.11	
Sigal et al, 2007	112	24	108	24	110	24	107	24
Sillanpaa et al, 2008	-1.8		2.173		-2.7		2.299	
Swift et al, 2012	-2.49		4.31		-1.76		3.968	
Zhou et al, 2022 a	90.93	12.5	87.5	12.5	93.43	9.06	91.87	8.12
Zhou et al, 2022 b	91.56	8.12	87.81	7.81	93.43	9.06	91.87	8.12

Supplementary Table S17. Means and SD or Mean difference and SD (main or estimated) for lean body mass in CT vs. RT

Study name	CT				AT			
	Pre		Post		Pre		Post	
	Mean	SD	Mean	SD	Mean	SD	Mean	SD
	Mean change		SD change		Mean change		SD change	
Ahtiainen et al, 2009	60	3	61	4	61	4	62	5
Church et al, 2010	0		0.21		0.8		1.92	
Cortz-Cooper et al. 2007	42.8	13.85	44.4	13.5	45	12.25	46.7	12.97
Irving et al, 2015	1.5		2.1		0.7		1.58	
Kim et al, 2018	19.33	2.95	18.83	2.95	20.39	2.44	20.05	2.83
Kobayashi et al, 2023	-0.23		1.22		0.3		1.24	
Schroeder et al, 2019	0.8		1.4		0.1		0.29	
Sigal et al, 2007	63.9	13.6	63.2	13.6	62.3	13.6	62.5	13.6
Sillanpaa et al, 2008	1.6		2.4		1.8		2.9	
Sillanpaa et al, 2010	41.433	3.495	42.299	3.204	40.893	4.523	41.212	4.605
Swift et al, 2012	0.04		2.057		0.92		1.875	
Timmons et al, 2018	0.4		0.87		-0.1		0.32	

Supplementary Table S18. Means and SD or Mean difference and SD (main or estimated) for muscle mass/volume in CT vs. RT

Study name	CT				AT			
	Pre		Post		Pre		Post	
	Mean	SD	Mean	SD	Mean	SD	Mean	SD
	Mean change		SD change		Mean change		SD change	
Aminilari et al, 2017	40.18	3.86	41.72	4.06	41.75	3.95	43.47	5.17
Campos et al, 2013 a (AS)	28.2	13.4	29.3	12.5	13.6	4.8	14.5	6.5
Campos et al, 2013 b (SA)	15.7	3.9	19.2	2.2	13.6	4.8	14.5	6.5
Chen et al. 2017	20.7	4	20.9	3.7	22.9	4	23.1	4
Davidson et al, 2009	0.62		0.86		0.97		1.09	
Ruangthai et al, 2019	21.9	5.1	22.1	5	18.1	2.2	18.2	2.1
Feiereisen et al, 2007	7121	1437	7449	1388	7054	1100	7328	1199

Supplementary Table S19. Means and SD or Mean difference and SD (main or estimated) for CSA in CT vs. RT

Study name	CT				AT			
	Pre		Post		Pre		Post	
	Mean	SD	Mean	SD	Mean	SD	Mean	SD
	Mean change		SD change		Mean change		SD change	
Ahtiainen et al, 2009	5021	829	5654	2214	4922	691	5913	877
Irving et al, 2015	5.1		8.4		5.6		10.75	
Izquterdo et al, 2004	39.61	4.8	43.49	5.42	46.06	10.79	51.48	13.86
Sigal et al, 2007	309	71	317	71	302	69	308	69
Karavirta et al, 2011	8.46		34.88		25.89		21.8	

Supplementary Table S20. Checklist item

Section and Topic	Item #	Checklist item	Location where item is reported
**TITLE**			
Title	1	Identify the report as a systematic review.	Page 1
**ABSTRACT**			
Abstract	2	See the PRISMA 2020 for Abstracts checklist.	Page 1
**INTRODUCTION**			
Rationale	3	Describe the rationale for the review in the context of existing knowledge.	Page 2
Objectives	4	Provide an explicit statement of the objective(s) or question(s) the review addresses.	Page 2
**METHODS**			
Eligibility criteria	5	Specify the inclusion and exclusion criteria for the review and how studies were grouped for the syntheses.	Page 3
Information sources	6	Specify all databases, registers, websites, organisations, reference lists and other sources searched or consulted to identify studies. Specify the date when each source was last searched or consulted.	Page 3
Search strategy	7	Present the full search strategies for all databases, registers and websites, including any filters and limits used.	Page 3
Selection process	8	Specify the methods used to decide whether a study met the inclusion criteria of the review, including how many reviewers screened each record and each report retrieved, whether they worked independently, and if applicable, details of automation tools used in the process.	Page 3
Data collection process	9	Specify the methods used to collect data from reports, including how many reviewers collected data from each report, whether they worked independently, any processes for obtaining or confirming data from study investigators, and if applicable, details of automation tools used in the process.	Page 4
Data items	10a	List and define all outcomes for which data were sought. Specify whether all results that were compatible with each outcome domain in each study were sought (e.g. for all measures, time points, analyses), and if not, the methods used to decide which results to collect.	Page 4
	10b	List and define all other variables for which data were sought (e.g. participant and intervention characteristics, funding sources). Describe any assumptions made about any missing or unclear information.	Page 4
Study risk of bias assessment	11	Specify the methods used to assess risk of bias in the included studies, including details of the tool(s) used, how many reviewers assessed each study and whether they worked independently, and if applicable, details of automation tools used in the process.	Page 4
Effect measures	12	Specify for each outcome the effect measure(s) (e.g. risk ratio, mean difference) used in the synthesis or presentation of results.	Page 4
Synthesis methods	13a	Describe the processes used to decide which studies were eligible for each synthesis (e.g. tabulating the study intervention characteristics and comparing against the planned groups for each synthesis (item #5)).	Page 4 and 5
	13b	Describe any methods required to prepare the data for presentation or synthesis, such as handling of missing summary statistics, or data conversions.	Page 4 and 5
	13c	Describe any methods used to tabulate or visually display results of individual studies and syntheses.	Page 4 and 5

Supplementary Table S20. Checklist item

Section and Topic	Item #	Checklist item	Location where item is reported
	13d	Describe any methods used to synthesize results and provide a rationale for the choice(s). If meta-analysis was performed, describe the model(s), method(s) to identify the presence and extent of statistical heterogeneity, and software package(s) used.	Page 4 and 5
	13e	Describe any methods used to explore possible causes of heterogeneity among study results (e.g. subgroup analysis, meta-regression).	Page 4 and 5
	13f	Describe any sensitivity analyses conducted to assess robustness of the synthesized results.	Page 4 and 5
Reporting bias assessment	14	Describe any methods used to assess risk of bias due to missing results in a synthesis (arising from reporting biases).	Page 4
Certainty assessment	15	Describe any methods used to assess certainty (or confidence) in the body of evidence for an outcome.	Page 4
**RESULTS**			
Study selection	16a	Describe the results of the search and selection process, from the number of records identified in the search to the number of studies included in the review, ideally using a flow diagram.	Page 5 and figure 1
	16b	Cite studies that might appear to meet the inclusion criteria, but which were excluded, and explain why they were excluded.	Page 5 and figure 1
Study characteristics	17	Cite each included study and present its characteristics.	Page 5 and 6 and table 1 and 2
Risk of bias in studies	18	Present assessments of risk of bias for each included study.	S table 1
Results of individual studies	19	For all outcomes, present, for each study: (a) summary statistics for each group (where appropriate) and (b) an effect estimate and its precision (e.g. confidence/credible interval), ideally using structured tables or plots.	Page 16-19
Results of syntheses	20a	For each synthesis, briefly summarise the characteristics and risk of bias among contributing studies.	Page 16-19
	20b	Present results of all statistical syntheses conducted. If meta-analysis was done, present for each the summary estimate and its precision (e.g. confidence/credible interval) and measures of statistical heterogeneity. If comparing groups, describe the direction of the effect.	Page 16-19
	20c	Present results of all investigations of possible causes of heterogeneity among study results.	Page 16-19
	20d	Present results of all sensitivity analyses conducted to assess the robustness of the synthesized results.	Page 16-19
Reporting biases	21	Present assessments of risk of bias due to missing results (arising from reporting biases) for each synthesis assessed.	Page 16-19
Certainty of evidence	22	Present assessments of certainty (or confidence) in the body of evidence for each outcome assessed.	Page 16-19
**DISCUSSION**			

Supplementary Table S20. Checklist item

Section and Topic	Item #	Checklist item	Location where item is reported
Discussion	23a	Provide a general interpretation of the results in the context of other evidence.	Page 27-29
	23b	Discuss any limitations of the evidence included in the review.	Page 27-29
	23c	Discuss any limitations of the review processes used.	Page 27-29
	23d	Discuss implications of the results for practice, policy, and future research.	Page 27-29
**OTHER INFORMATION**			
Registration and protocol	24a	Provide registration information for the review, including register name and registration number, or state that the review was not registered.	Page 2
	24b	Indicate where the review protocol can be accessed, or state that a protocol was not prepared.	Page 2
	24c	Describe and explain any amendments to information provided at registration or in the protocol.	Page 2
Support	25	Describe sources of financial or non-financial support for the review, and the role of the funders or sponsors in the review.	Page 29
Competing interests	26	Declare any competing interests of review authors.	Page 29
Availability of data, code and other materials	27	Report which of the following are publicly available and where they can be found: template data collection forms; data extracted from included studies; data used for all analyses; analytic code; any other materials used in the review.	Page 29

From: Page MJ, McKenzie JE, Bossuyt PM, Boutron I, Hoffmann TC, Mulrow CD, et al. The PRISMA 2020 statement: an updated guideline for reporting systematic reviews. BMJ 2021;372:n71. doi: 10.1136/bmj.n71. This work is licensed under CC BY 4.0. To view a copy of this license, visit https://creativecommons.org/licenses/by/4.0/
